# Development of a multi-neoepitope vaccine targeting non-small cell lung cancer through reverse vaccinology and bioinformatics approaches

**DOI:** 10.3389/fimmu.2025.1521700

**Published:** 2025-05-16

**Authors:** Elahe Asadollahi, Alireza Zomorodipour, Zahra-Soheila Soheili, Babak Jahangiri, Majid Sadeghizadeh

**Affiliations:** ^1^ Department of Molecular Genetics, Faculty of Biological Sciences, Tarbiat Modares University, Tehran, Iran; ^2^ Department of Molecular Medicine, Institute of Medical Biotechnology, Institute of Genetic Engineering and Biotechnology, Tehran, Iran

**Keywords:** NSCLC, immunotherapy, targeted treatments, tumor-specific antigens, neoantigens, reverse vaccinology, multi-neoepitope vaccine, cancer vaccine

## Abstract

**Introduction:**

Lung cancer, predominantly non-small cell lung cancer (NSCLC), is the leading cause of cancer-related mortality worldwide. Among immunotherapeutic strategies, the personalized multi-neoepitope vaccine (MNEV) offers a promising approach for managing advanced-stage NSCLC.

**Methods:**

We used reverse vaccinology, immunoinformatics, and bioinformatics to design an MNEV targeting lung cancer in murine (LL/2) cells. Whole exome sequencing (WES) and RNA sequencing data from human and mouse NSCLC cell lines were analyzed to select neoantigens, which were evaluated for their ability to stimulate B cells, helper T lymphocytes (HTLs), and cytotoxic T lymphocytes (CTLs). Molecular docking studies estimated the binding affinity of mouse neoepitopes with MHC class I, MHC class II, and B-cell receptors. Suitable linkers were selected to construct the MNEV, with the 50S L7/L12 ribosomal protein sequence included as an adjuvant to enhance immune responses. The immunoglobulin kappa (Igκ) chain signal peptide was incorporated to improve secretion efficiency. The stability of the final MNEV construct in complex with TLR3, TLR4, and TLR9 was confirmed through binding analysis and refinement of the best-predicted 3D model. To evaluate the immunological efficacy of the MNEV, female C57BL/6 mice were immunized subcutaneously. Immune responses were assessed by measuring total IgG levels in serum using enzyme-linked immunosorbent assay (ELISA) and quantifying IFN-γ and granzyme B levels in the supernatant of cultured splenocytes. The proportions of CD19+ B cells and CD4+ and CD8+ T cells were determined using flow cytometric analysis.

**Results:**

In silico evaluations indicated that the MNEV is non-toxic, non-allergenic, and stable, exhibiting high-affinity interactions with B lymphocytes, CTLs, and HTLs. Immunization with the MNEV significantly increased serum IgG levels. Flow cytometry analysis revealed higher percentages of CD19+ B cells and CD4+ and CD8+ T cells. Furthermore, splenocytes from immunized mice showed a marked increase in IFN-γ and granzyme B secretion compared to control groups.

**Discussion:**

This study demonstrates that the MNEV induces a robust strong immune response, highlighting its potential as a promising approach for cancer prevention and immunotherapy, particularly for NSCLC. Furthermore, it provides a foundation for developing neoepitope-based vaccines against various malignancies, guiding future research in cancer vaccine development through advanced computational methods in immunology and oncology.

## Graphical Abstract

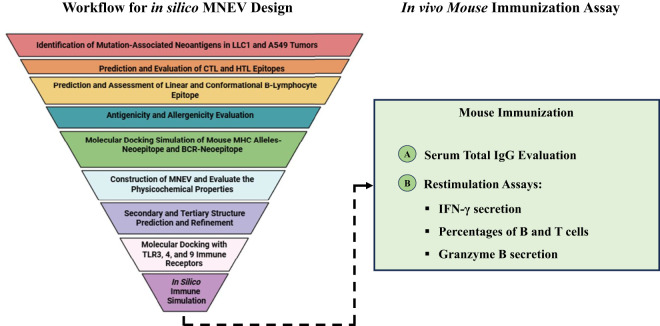

## Introduction

Lung cancer, predominantly non-small cell lung cancer (NSCLC), which constitutes approximately 85% of cases, remains the leading cause of cancer-related mortality globally, claiming around 1.8 million lives in 2020 (1–3). NSCLC originates from genetic and epigenetic alterations that drive uncontrolled proliferation of somatic cells (4, 5). Current treatment modalities—surgery, radiation, chemotherapy, targeted therapies, and immunotherapy—are hindered by significant limitations, including their inapplicability to advanced-stage disease, toxicity, emergence of resistance, and efficacy restricted to specific patient cohorts (6–9). These challenges are exacerbated by tumor heterogeneity, resistance mechanisms involving secondary mutations or alternative pathways, and a scarcity of reliable biomarkers. Consequently, there is a pressing need for innovative therapeutic strategies. Neoantigen-based vaccines offer a promising alternative by targeting personalized tumor mutations, enhancing specificity and safety while potentially minimizing off-target effects and resistance (10, 11). Current neoantigen vaccines for NSCLC demonstrate potential in preclinical models and clinical trials, though challenges remain. For instance, a phase I trial of personalized neoantigen peptide vaccine (PPV) in advanced NSCLC patients established safety and feasibility, yielding notable clinical outcomes, including one complete response and six partial responses, particularly among EGFR-mutated patients (12). By eliciting robust, patient-specific T-cell responses, this approach could improve NSCLC outcomes, addressing both the limitations of conventional therapies and disparities arising from costly treatments (13).

NSCLC presents distinct therapeutic hurdles, particularly with immunotherapy, where patient responses vary widely due to the limited predictive power of biomarkers such as PD-L1 expression and tumor mutation burden (TMB) (14, 15). Currently, only 15-20% of patients respond effectively to PD-1 inhibitors without prior selection, emphasizing the need for reliable indicators to identify suitable candidates (14, 16). Additionally, frequent treatment resistance undermines the long-term efficacy of these therapies. Neoantigen-based vaccines, however, target tumor-specific antigens arising from cancer cell mutations, potentially overcoming these shortcomings. Additionally, the NEO-PV-01 vaccine, which combines personalized neoantigens with anti-PD-1 therapy, has shown promise in early-phase trials by inducing CD4+ and CD8+ T-cell responses (17). Studies demonstrate their ability to provoke vigorous T-cell activation, offering a personalized strategy that enhances treatment efficacy (18, 19). Moreover, these vaccines can be designed to stimulate both CD4+ and CD8+ T-cell responses, bolstering their effectiveness against NSCLC (20). However, many studies, including those evaluating PPV and NEO-PV-01, involve small patient cohorts, underscoring the need for larger trials to confirm efficacy and safety (13, 21). This study aims to leverage these advantages to develop more efficacious treatment options for NSCLC patients while addressing persistent limitations through further research.

Cancer cells, during their evolution, accumulate numerous mutations, some of which produce neoantigens—unique antigens critical to tumor survival and recognized as non-self by the immune system (22). T cells can target these neoantigens to attack and eliminate tumors. Neoantigen-based subunit vaccines, considered among the most advanced cancer therapies developed in recent decades, exploit this mechanism (23, 24). A novel approach, reverse vaccinology, integrates immunogenomics and bioinformatics to accelerate vaccine development, reducing both time and cost compared to traditional methods (25, 26). Computational tools, including protein modeling, epitope prediction, and protein-protein interaction analysis, facilitate in-silico vaccine design by processing vast immunological datasets, such as antigen presentation profiles (27–29).

Next-generation therapeutic vaccines harness the immune system to combat cancer, with multi-epitope designs playing a pivotal role. The selection of adjuvants, linkers, antigens, and epitopes critically influences clinical outcomes (30). To streamline development, which is typically costly and protracted, various strategies have been implemented to enhance efficiency (31). Effective peptide-based vaccines rely on immunodominant B-cell and T-cell responses to generate robust, lasting immunity (32). Recent advances in multi-epitope vaccine design have incorporated B-cell, CD8+ cytotoxic T-lymphocyte (CTL), and CD4+ helper T-lymphocyte (HTL) epitopes (33). In cancer immunotherapy, MNEVs provide broad protection against tumor heterogeneity, personalized treatment tailored to individual mutation profiles, and improved specificity and safety by selectively targeting malignant cells (34, 35). These vaccines can be combined with therapies like checkpoint inhibitors to enhance efficacy and induce durable immune responses (36, 37). While neoantigen vaccines show promise as a novel NSCLC treatment, further research is essential to optimize their clinical application and overcome current limitations, such as small trial sizes and variable patient responses. Looking ahead, expanding their application across cancer types, leveraging cutting-edge technologies, and optimizing production processes could broaden access to these tailored therapies. Ongoing research and clinical trials suggest that MNEVs hold significant promise as integral components of future cancer treatment strategies.

Despite the potential of neoantigen-based vaccines to address NSCLC’s therapeutic challenges, including tumor heterogeneity, immunotherapy resistance, and limited biomarker reliability, their clinical translation remains constrained by small-scale trials, variable efficacy, and the complexity of tailoring treatments to individual mutation profiles (13, 21). These limitations highlight an unmet need for scalable, broadly effective vaccine designs that can consistently elicit robust immune responses across diverse patient populations. This study addresses these challenges by developing a MNEV for NSCLC, using reverse vaccinology and bioinformatics to identify and design neoantigens that elicit robust B-cell, CD4+, and CD8+ T-cell responses. By integrating advanced computational strategies with experimental validation in preclinical models, this work aims to enhance the specificity, safety, and immunogenicity of NSCLC-targeted vaccines, offering a foundation for next-generation immunotherapies that could improve patient outcomes and extend the applicability of personalized cancer treatments.

## Materials and methods

### Identification of mutation-associated neoantigens in LL/2 (LLC1) and A549 tumors

Raw exome sequencing data from the Illumina^®^-sequenced NSCLC carcinoma experiment, specifically from LL/2 and A549 cells, are accessible through the following databases: PRJNA758177 and PRJNA603489). Furthermore, raw RNA sequencing data (sequenced utilizing Illumina^®^ technology) from murine LL/2 (NCBI Bioproject studies PRJNA759882, PRJNA401728, and PRJNA786566) and A549 (NCBI Bioproject study PRJNA1201561) NSCLC cell lines were obtained from the Sequence Read Archive (SRA). FastQC was used for quality control, while Trimmomatic v0.38 was used for read trimming, including adapter removal and low-quality base filtering (parameters: SLIDINGWINDOW:4:30, TRAILING:30, LEADING:30, HEADCROP:15, MINLEN:40) (38). Paired-end reads were mapped to the mouse (GRCm38/mm10) and human (GRCh38/hg38) reference genomes using BWA-MEM (v0.7.17) for whole exome sequencing (WES) data and STAR V.2.7.0 for RNA-seq data, with soft-clipping enabled by default and a minimum mapping quality threshold of 30. Sambamba (v0.6.8) was used for duplicate removal as part of the post-alignment processing (39), while Samtools (v1.15) was used for standard data manipulation, such as sorting, BAM generation, and indexing. Whole-exome sequencing data were then analyzed for somatic single nucleotide variations (SNVs). Somatic variant calling was performed using MuTect (1.1.7) (40), and results were combined by taking a union of called variants. RNA-Seq data were used to quantify tumor-specific non-synonymous variant expression using FPKM values. We employed isovar (protein-sequence-length 30) for Cancer Neoantigen Prediction (41). Another benefit is that isovar automatically predicts allele-specific expression by identifying each mutant protein sequence using mutation-supporting RNA readings. Mutant candidates with FPKM >1 were selected. MuTect2 intrinsic filters, read orientation artifact filters, and strain-specific polymorphism filters were used to further filter the variants. Variant Effect Predictor (VEP) (https://useast.ensembl.org/info/docs/tools/vep/script/vep_options.html) was used to annotate variants that passed all criteria. **Supplementary Data S1** and **S2** provide Variant Call Format (VCF) files and isovar results.

### Epitope prediction and designing of the MNEV

#### Prediction and evaluation of CTL and HTL epitopes

Initially, we selected expressed mutations with FPKM ≥ 1. CTL epitopes from antigenic and non-allergenic proteins were identified from mouse MHC Class I (MHC I) haplotypes (H2Kb, H2Db) and human haplotypes (HLA-A30/HLA-B44) using four distinct algorithms: NetMHCpan4.1 (42), NetCTLpan, NetMHCcons (43), and SYFPEITHI (44). For human MHC I, we applied specific criteria: NetMHCPan 4.1 required a binding affinity < 500 nM and a % rank < 0.5; NetMHCcons required an affinity < 500 nM; SYFPEITHI required a score >12; NetCTLpan required a TAP score >0.5 and a C-terminal cleavage score >0.5. According to the NetMHCpan algorithm (BA) available at http://www.cbs.dtu.dk/services/NetMHCpan/, accessed on February 7, 2019, epitopes with binding affinities <500 nM and % ranks <0.5 were selected (45). As of February 8, 2019, the acceptability criteria for the SYFPEITHI server at http://www.syfpeithi.de/bin/MHCServer.dll/EpitopePrediction.htm were set at scores greater than or equal to >12 for prediction accuracy (46). The accuracy of other techniques was complemented by NetMHCcons, which combines two MHC-peptide binding methods: NetMHCpan and Pickpocket (43). We chose sequences located within identified cleavage sites that had peptides with lengths between nine to eleven residues, IC50 values <500 nmol/dm³, proteasome-processing scores >0.5, and TAP transport efficiency scores >0.5. To account for antigen processing and presentation accurately, we used the NetCTLpan server available at https://services.healthtech.dtu.dk/service.php?NetCTLpan-1.1 to combine predictions of MHC I binding affinities along with TAP transport efficiency and proteasomal C-terminal cleavage predictions. NetMHCcons was used to complement other tools by integrating NetMHCpan and Pickpocket for improved MHC-peptide binding prediction. We selected sequences that are not located within identified cleavage sites, focusing on peptides with lengths between nine to eleven residues, IC50 values less than 500 nmol/L, proteasome-processing scores greater than 0.5, and TAP transport efficiency scores greater than 0.5 (47). To account for antigen processing and presentation accurately, we utilized the NetCTLpan server available at https://services.healthtech.dtu.dk/service.php?NetCTLpan-1.1. This server combines predictions of MHC I binding affinities along with TAP transport efficiency and proteasomal C-terminal cleavage predictions (48).

The CD4+ T cell epitopes were selected with lengths of 9–22 amino acids. Here, we investigate the efficacy of several approaches for integrating five servers for the prediction of MHC Class II (MHC II) binding for the mouse allele (H2-IAb): RANKPEP (threshold score of top 2%) (49), IEDB (percentile > 0.10) (50), MHC2PRED (scored > 0.2) (http://webs.iiitd.edu.in/raghava/mhc2pred/), and NetMHCIIpan-4.0 (BA, SMM-Align; IC50 <500 nM, Percentile Rank ≤10) (25) and NetMHCII-1.1 (SMM-Align; Adjusted Rank <10) (www.cbs.dtu.dk/services/NetMHCII-1.1/). In order to forecast how a certain peptide sequence would attach to the MHC II molecule, NetMHCIIpan-4.0 uses neural network technology (51). In order to provide more accurate and dependable overall forecasts than when utilized separately, our goal is to test their combined usage. Predictions were combined using union (including all binders predicted by any server) and intersection (selecting only binders predicted by all servers) modes to balance sensitivity and specificity. The most common MHC II alleles were used to obtain HTL epitopes (52), and epitopes with an IC50 <500 nM are considered excellent binders (53). We investigated the efficacy of several approaches for integrating predictions from three servers to predict MHC II binding for specific human alleles (HLA-DRB1*04:04, HLA-DRB1*04:01, and HLA-DRB1*07:01). The servers used included NetMHCIIpan-4.1 BA (with criteria IC50 < 500 nM and Percentile Rank ≤ 10), NetMHCII 1.1 (SMM-align) with criteria IC50 < 500 nM and Adjusted Rank < 5, and CONSENSUS 2.2 with an Adjusted Rank < 10.

Consensus sequences were assessed, resulting in 15–17 remaining sequences that passed all rounds of evaluation for toxicity, allergies, and antigenicity. It is critical to recognize that different MHC II binders can induce the production of different types of cytokines. To predict IL-4-inducing peptides, we used IL4pred (http://crdd.osdd.net/raghava/il4pred/). For the prediction of IFN-gamma-inducing peptides, we utilized the IFNepitope webserver (http://crdd.osdd.net/raghava/ifnepitope/index.php). Both servers employ prediction models based on Support Vector Machines (SVM), with the SVM threshold and other parameters set to their default values. For the prediction of IL-10-inducing peptides, we utilized IL-10Pred (https://webs.iiitd.edu.in/raghava/il10pred/predict3.php), with the SVM threshold set to -0.3. (Supplementary data are presented in **Table 1**; **Supplementary Data S3**).

#### Prediction and assessment of linear and conformational B-lymphocyte epitope

Linear B-cell epitopes were predicted using ABCpred with a threshold of 0.51 and window length 16 (http://www.imtech.res.in/raghava/abcpred/) and BepiPred with a threshold of 0.35 (http://www.cbs.dtu.dk/services/BepiPred. These two software types, which are now the most widely used online servers for assessing Linear B epitopes of anticipated proteins, rely on distinct databases and algorithms (54, 55). The best dominant linear B-cell epitopes of proteins are confirmed by overlapping predictions from ABCpred and BepiPred. This can significantly increase the accuracy of epitope prediction.

Effective anti-tumor immunity also depends on the interaction of antibodies with linear and conformational B-lymphocyte epitopes. Protein three-dimensional (3D) structures are necessary for all conformational epitope prediction techniques. Using the ElliPro service (56), with a minimum score of 0.5 and a maximum distance of 6 Ångströms, Conformational (discontinuous) epitopes in mutant proteins were predicted. ElliPro was chosen for its robust prediction of conformational epitopes, with an AUC of 0.732, indicating moderate to high accuracy compared to other tools. This tool utilizes protein-antibody interactions to predict discontinuous epitopes. The IgPred module (>0.5) (57) (https://webs.iiitd.edu.in/raghava/igpred/index.html) was created to forecast which B-cell epitopes would produce which antibody classes. IgPred allowed us to determine the epitope’s propensity to elicit IgG antibodies (**Table 1**; **Supplementary Data S3**).

Table 1Neoepitopes prediction and prioritization.

**Table:** 

MHC I									
Neoepitope	Allele	NetMHCPan 4.1 (BA)	SYFPEITHI	NetMHCcons	NetCTLpan	NetCTLpan	Immunogenicity	Allergen	IFN-γ Response
		Aff (nM) <500nM/%Rank <0.5	>12	Affinity (nM)<500nM/PROTEASOM SCORE>0.5)	C-terminal cleavage (Cle) score >0.5	TAP score > 0.5	VaxiJen v2.0 (Threshold > 0.4)	Allertop v2.0	IFNepitope
LL/2 (LLC1) cell line									
SLYTEYWKLLR	H-2-Kb	257.89/0.33	23	231/0.98	0.55544	0.918	1.0642 (Probable ANTIGEN).	non-allergen	POSITIVE
IAHEDYMEL	H-2-Kb	319.78/0.39	16	418.3/1.40	0.97386	1.058	0.7680 (Probable ANTIGEN).	non-allergen	POSITIVE
VSFQNQLTNWL	H-2-Db	102.36/0.05	16	276.1/1.36	0.76833	1.316	0.6731 (Probable ANTIGEN)	non-allergen	POSITIVE
VATDYLVGI	H-2-Kb	427.81/0.5	12	442.1/1.32	0.83622	0.582	0.4008 (Probable ANTIGEN).	non-allergen	POSITIVE
FGLINVTPNML	H-2-Db	25.59/0,03	18	369.8/1.53	0.95841	0.528	0.4808 (Probable ANTIGEN).	non-allergen	NEGATIVE
A549 cell line									
Neoepitope	Allele	NetMHCPan 4.1 (BA)	SYFPEITHI	NetMHCcons	NetCTLpan		Immunogenicity	Allergen	IFN-γ Response
		Aff(nM)<500nM/%Rank<0.5	>12	Affinity (nM)<500nM/PROTEASOM SCORE>0.5	TAP score (Cut off) > 0.5	C-terminal cleavage (Cle) score >0.5	VaxiJen v2.0 (Threshold > 0.4)	Allertop v2.0	IFNepitope
KGDRSSLYLV	HLA-A*30:01	85.54/0.41	12	130.18	2.918	0.9379	0.8103 (Probable ANTIGEN).	Probable NON-ALLERGEN	NEGATIVE
SSKGDRSSLY	HLA-A*30:02	87.72/0.14	13	35.73	2.918	0.9379	1.4106 ((Probable ANTIGEN).	Probable NON-ALLERGEN	NEGATIVE
SLLSSKGDRSSLY	HLA-A*30:02	229.35/0.4	25	42.94	1.094	0.9683	1.1868 (Probable ANTIGEN).	Probable NON-ALLERGEN	POSITIVE
AERAASPQPGPW	HLA-B*44:02 HLA-B*44:03	95.49/0.09 181.63/0.14	18	46.07 95.12	1.209	0.9537	0.8160 (Probable ANTIGEN).	Probable NON-ALLERGEN	POSITIVE
RLRSGAHVVV	HLA-A*30:01	36.34/0.17	14	122.66	0.653	0.9747	0.5290 (Probable ANTIGEN).	Probable NON-ALLERGEN	POSITIVE
RVRTLSGSRPPL	HLA-A*30:01	45.68/0.22	24	6.43	1.081	0.966	0.9655 (Probable ANTIGEN).	Probable NON-ALLERGEN	POSITIVE
KSFPISWDAY	HLA-A*30:02	13.54/0.02	13	27.41	3.231	0.921	0.7151 (Probable ANTIGEN).	Probable NON-ALLERGEN	POSITIVE

**Table:** 

MHC II											
Epitope	Allele	RANKPEP (Threshold score> 2%.)	NetMHCIIpan-4.0 BA (IC50/Percentile Rank ≤ 10)	IEDB (percentile> 0.10)	MHC2pred (scored > 0.2)	NetMHCII 1.1 (SMM -Align) (Adjusted rank<10)	Immunogenicity VaxiJen v2.0 (Threshold > 0.4)	Allergen (Allertop v2.0)	IFN-γ	IL-4	IL-10
LL/2 (LLC1) cell line											
GPSYFKSSASVTGEP	I-Ab	3.746	235.53/0.74	2.71	0.232	4.6	0.4929 (Probable ANTIGEN).	non-allergen	POSITIVE	IL4-inducer	IL10 inducer
KYSSARAVRMPRHEKSP	I-Ab	11.713	184.2/0.5	18.3	0.776	9	0.8199 (Probable ANTIGEN).	non-allergen	NEGATIVE	Non-IL4-inducer	IL10 inducer
GVADFHYAASKALRV	I-Ab	12.419	37.12/0.01	0.12	0.277	0.01	0.6291 (Probable ANTIGEN).	non-allergen	NEGATIVE	IL4-inducer	Non IL10 inducer
TGVADFHYAASKALR	I-Ab	8.386	208.09/0.59	0.22	0.312	0.01	0.5927 (Probable ANTIGEN).	non-allergen	POSITIVE	IL4-inducer	Non IL10 inducer
A549 cell line											
	Allele	NetMHCIIpan-4.0 BA (IC50/Percentile Rank ≤ 10)		NetMHCII 1.1 (SMM -Align) (IC50<500nM/Adjusted rank<5)		CONSENSUS 2.2 (Adjusted rank<10)	Immunogenicity VaxiJen v2.0 (threshold > 0.4)	Allergen (Allertop v2.0)	IFN-γ	IL-4	IL-10
IAALHHFYSKHLGFP	HLA-DRB1*07:01	72.59/6.40		30.00/1.8		1.8	0.6243 (Probable ANTIGEN).	Probable NON-ALLERGEN	POSITIVE	IL4-inducer	IL10 inducer
RWFEFLHPGQVYRLV	HLA-DRB1*07:01	50.04/4.20		68.00/4.60		7.2	1.2316 (Probable ANTIGEN).	Probable NON-ALLERGEN	POSITIVE	IL4-inducer	IL10 inducer
QLSQALSLMETVKQG	HLA-DRB1*04:01	116.70/4.90		129.0/2.50		2.7	0.9110 (Probable ANTIGEN).	Probable NON-ALLERGEN	POSITIVE	IL4-inducer	IL10 inducer
LRWRLLQAQAAGVDW	HLA-DRB1*04:04	94.47/5.40		57.00/2.50		5.3	0.9382 (Probable ANTIGEN).	Probable NON-ALLERGEN	POSITIVE	IL4-inducer	IL10 inducer
SELKIMCTVDHQGQR	HLA-DRB1*04:04	64.82/3.20		20.00/0.5		0.5	0.9866 (Probable ANTIGEN).	Probable NON-ALLERGEN	POSITIVE	IL4-inducer	IL10 inducer

**Table:** 

Linear B-Lymphocyte (LBL)							
Epitope	ABCPred	BepiPred-2.0		SVMTriP (score)	Immunogenicity (VaxiJen v2.0) (Threshold > 0.4)	Allergen (Allertop v2.0)	IgPred module
LL/2 (LLC1) cell line							
GELECRSPPRMHGAKA	GELECRSP**P**RMHGAKA (0.88)	GELECRS**P**PRMHGAKA		GELECRSPP**R**MHGAKA (1)	0.7442 (Probable ANTIGEN).	non-allergen	IgG Epitope (0.513)
IDILQRRQEGQASKDP	IDILQRRQE**G**QASKDP (0.83)	IDILQRRQE**G**QASKDP		IDILQRRQEG**Q**ASKDP (1)	0.8143 (Probable ANTIGEN).	non-allergen	IgG Epitope (0.566 *)
ATSERKDMTFDTLRNR	ATSE**R**KDMTFDTLRNR (0.82)	ATSE**R**KDMTFDTLRNR		ATSE**R**KDMTFDTLRNR (1)	0.8725 (Probable ANTIGEN).	non-allergen	IgG Epitope (0.641 *)
FRDTQKKLEEEKGKKE	FRDT**Q**KKLEEEKGKKE (0.83)	FRDT**Q**KKLEEEKGKKE		FRDT**Q**KKLEEEKGKKE (1)	1.1644 (Probable ANTIGEN).	non-allergen	IgG Epitope (0.503)
A549 cell line							
	ABCPred (Threshold = 0.51) (window length to use for prediction=16)		BepiPred-2.0 (the threshold of 0.35)		Immunogenicity (VaxiJen v2.0 (Threshold > 0.4)	Allergen (Allertop v2.0)	IgPred module
AEAQNHNCVPDVKALM	AEAQNH**N**CVPDVKALM (0.83)		AEAQNH**N**CVPDVKALM		0.4773 (Probable ANTIGEN).	Probable NON-ALLERGEN	IgG Epitope (1.280 *)
ACLGSNRALFREPDLV	ACLGSNRALFR**E**PDLV (0.68)		**ACLGSNRALFREPDLV**		0.7252 (Probable ANTIGEN).	Probable NON-ALLERGEN	IgG Epitope (1.280 *)
GDSTGGRPRSRAVAST	GDSTGG**R**PRSRAVAST (0.86)		**GDSTGGRPRSRAVAST**		1.3495 (Probable ANTIGEN).	Probable NON-ALLERGEN	IgG Epitope (1.280 *)
WEKIASDLTRSQDLVI	WEKIASDLTRSQDL**V**I (0.83)		**WEKIASDLTRSQDLVI**		0.6263 (Probable ANTIGEN).	Probable NON-ALLERGEN	IgG Epitope (0.6)
TIEQKMADYSNKLYHQ	TIEQKMADYSNKLY**H**Q (0.84)		TIEQKMADYSNKLY**H**Q		0.6606 (Probable ANTIGEN).	Probable NON-ALLERGEN	IgG Epitope (0.577 *)
PVEQSAPDSGQANLTS	PVEQ**S**APDSGQANLTS (0.97)		**PVEQ**S**APDSGQANLTS**		0.4600 (Probable ANTIGEN).	Probable NON-ALLERGEN	IgG Epitope (0.788 *)
TFVPRWSPPSITPSSE	TFVPRWSPPSIT**P**SSE (0.81)		**TFVPRWSPPSITPSSE**		0.6605 (Probable ANTIGEN).	Probable NON-ALLERGEN	IgG Epitope (0.688 *)

**Table:** 

Conformational or discontinuous B cell epitopes						
PDB ID/Chain	ElliPro	EPITOP	Immunogenicity	Allergen	IgPred module	Score
LL/2 (LLC1) cell line						
5GS9/A	A:M1, A:E2, A:E7, A:S100, A:E107, A:H108, A:H109, A:L130, A:S131, A:V132, A:I134, A:H135, A:T136, A:L137, A:A138, A:Q139, A:E140, A:F141, A:D142, A:I143, A:Y144, A:E146, A:V147, **A:A148**, A:G149, A:E150, A:P151, A:V152, A:P153, A:V154, A:T155, A:R156, A:D157 (0.784)	MEESEHHLSVIHTLAQEFDIYEV**A**GEPVPVTRD	0.6333 (Probable ANTIGEN).	non-allergen	IgG Epitope (0.750 *)	0.784
4YFD/A	B:P242, B:P243, B:S247, B:P248, B:N249, B:D250, B:R251, B:V252, B:V253, B:Y254, B:E255, B:K256, B:E257, B:P258, B:G259, B:E260, B:E261, B:L262, B:V263, B:I264, B:P265, B:C266, B:T283, B:I284, B:D285, B:G286, B:K287, B:K288, B:P289, B:D290, B:D291, B:V292, B:T293, B:V294, B:D295, B:I296, B:T297, B:L316, B:S317, B:I318, B:K319, B:K320, B:V321, B:T322, B:P323, B:E324, B:D325, B:L326, B:R328, B:N329, B:Y330, B:V331, B:H333, B:K338, B:G339, B:E340, B:A341, **B:Q342**, B:Q343, B:A344, B:A345	PPSPNDRVVYEKEPGEELVIPCTIDGKKPDDVTVDITLSIKKVTPEDLRNYVHKGEA**Q**QAA	0.5275 (Probable ANTIGEN).	non-allergen	IgG Epitope (1.494 *)	0.763
2I7V/A	A:E8, A:S9, A:D10, A:Q11, A:L12, A:L13, A:Q21, A:E31, A:F32, A:K33, A:G34, A:R35, A:K36, A:P54, A:Y55, A:D57, A:L58, A:I59, A:D60, A:P61, A:A62, A:E63, A:I64, A:D65, A:W82, A:L84, A:Q85, A:K86, A:T87, A:S88, A:F89, A:K90, A:G91, A:R92, A:Y123, A:T124, A:E125, A:T126, A:D127, A:L128, A:E129, A:E130, A:S131, A:M132, A:D133, A:K134, A:E136, A:T137, A:E145, A:V146, A:A147, **A:V148**, A:I149, A:K150, A:E168, A:I169, A:A170, A:G171, A:V172, A:K173, A:E192, A:I193, A:P194, A:N195, A:I196, A:K197, A:P198, A:D199, A:G208, A:T209, A:H210, A:Y399, A:Q400, A:S403, A:E404, A:R407, A:A408, A:L409, A:K410, A:P411, A:P412, A:H413, A:Q421, A:N422, A:A425, A:R426, A:K428, A:A429, A:A430, A:L431, A:I432, A:R433, A:E434, A:Y435, A:E436, A:D437, A:N438, A:D439, A:V441, A:H442, A:I443, A:E444, A:V445, A:N447, A:P448, A:R449, A:N450, A:T451, A:E452, A:A453, A:V454, A:T455, A:L456, A:N457, A:F458	ESDQLLQEFKGRKPYDLIDPAEIDWLQKTSFKGRYTETDLEESMDKETEVA**V**IKEIAGVKEIPNIKPDGTHYQSERALKPPHQNARKAALIREYEDNDVHIEVNPRNTEAVTLNF	0.5633 (Probable ANTIGEN).		IgG Epitope (1.453 *)	0.693
3IEG/A	A:S274, A:E277, A:L278, A:R280, A:D281, A:G282, A:R283, A:Y284, A:T285, A:D286, A:S289, A:K290, A:E292, A:S293, A:K296, A:T297, A:D319, A:E320, A:K321, A:P322, A:V323, A:E324, A:R327, A:A347, A:E348, A:A349, A:Y350, A:L351, A:I352, A:E353, A:E354, A:M355, A:Y356, A:D357, A:E358, A:A359, A:I360, A:Q361, A:D362, A:Y363, A:E364, A:A365, A:Q367, A:E368, A:H369, A:N370, A:E371, A:N372, **A:A373**, A:Q374, A:Q375, A:I376, A:R377, A:E378, A:G379, A:L380, A:E381, A:K382, A:A383, A:Q384, A:R385, A:L386, A:L387, A:K388, A:Q389, A:S390, A:Q391	SELRDGRYTDSKESKTDEKPVERAEAYLIEEMYDEAQEHNEN**A**QQIREGLEKAQRLLKQSQ	0.7494 (Probable ANTIGEN).	non-allergen	IgG Epitope (0.956 *)	0.735
A549 cell line						
PDB ID/Chain	ElliPro	EPITOP	Immunogenicity	Allergen	IgPred module	Score
1NM8/A	A:R90, A:K105, A:Q106, A:D107, A:F108, A:V109, A:D110, A:L111, A:Q112, A:G113, A:L115, A:R116, A:K120, A:E123, A:P162, A:G163, A:P164, A:K165, A:Q166, A:S173, A:K174, A:T175, A:K176, A:K177, A:P178, A:P179, A:T180, A:N187, A:Y188, A:Q189, A:E192, A:D194, A:V195, A:Y196, A:H197, A:S198, A:D199, A:G200, A:T201, A:P202, A:L203, A:T204, A:A205, A:D206, A:Q207, A:I208, A:F209, A:V210, A:Q211, A:L212, A:E213, A:K214, A:I215, A:W216, A:N217, A:S218, A:S219, A:L220, A:Q221, A:T222, A:N223, A:K224, A:E225, A:P226, A:N236, A:S237, A:A239, A:K240, A:A241, A:Y242, A:N243, A:T244, A:L245, A:I246, A:K247, A:D248, A:K249, A:V250, A:N251, A:R252, A:D253, A:S254, A:R256, A:A269, A:T270, A:M271, A:P272, A:R273, A:G292, A:S293, A:R294, A:G298, A:E312, A:D313, A:G314, A:S315, A:Y340, A:K343, A:P344, A:E345, A:L346, A:V347, A:R348, A:S349, A:P350, **A:L351**, A:V352, A:P353, A:L354, A:P355, A:M356, A:P357, A:K358, A:K359, A:L360, A:R361, A:F362, A:N363, A:I364, A:T365, A:P366, A:E367, A:I368, A:K369, A:S370, A:D371, A:I372, A:E373, A:K374, A:K376, A:Q377	RKQDFVDLQGLRKEPGPKQSKTKKPPTNYQEDVYHSDGTPLTADQIFVQLEKIWNSSLQTNKEPNSAKAYNTLIKDKVNRDSRATMPRGSRGEDGSYKPELVRSP**L**VPLPMPKKLRFNITPEIKSDIEKKQ	0.5551 (Probable ANTIGEN).	Probable NON-ALLERGEN	IgG Epitope (1.743 *)	0.702
8VVY/C	C:S13, C:T14, C:A15, C:A16, C:F17, C:H18, C:I19, C:S20, C:S21, C:L22, C:L23, C:E24, C:K25, C:M26, C:T27, C:S28, C:S29, C:D30, C:K31, C:D32, C:F33, C:R34, C:F35, C:M36, C:A37, C:T38, C:S39, C:D40, C:L41, C:M42, C:S43, C:E44, C:L45, C:Q46, C:K47, C:D48, C:S49, C:I50, C:Q51, C:L52, C:D53, C:E54, C:D55, C:S56, C:E57, C:R58, C:K59, C:V60, C:V61, C:K62, C:M63, C:L64, C:L65, C:R66, C:L67, C:L68, C:E69, C:D70, C:K71, C:N72, C:G73, C:E74, C:V75, C:Q76, C:N77, C:L78, C:A79, C:V80, C:K81, C:C82, C:L83, C:G84, C:P85, C:L86, C:V87, **C:A88**, C:K89, C:V90, C:K91, C:E92, C:Y93, C:Q94, C:V95, C:E96, C:T97, C:I98, C:V99, C:D100, C:T101, C:L102, C:C103, C:T104, C:N105, C:M106, C:R107, C:S108, C:D109, C:Q112, C:L113, C:R114, C:D115, C:I116, C:A117, C:G118, C:I119, C:G120, C:L121, C:V124, C:G136, C:L137, C:A138, C:T139, C:N140, C:V141, C:C142, C:R143, C:K144, C:I145, C:T146, C:G147, C:Q148, C:L149, C:T150, C:S151, C:A152, C:I153, C:A154, C:Q155, C:Q156, C:E157, C:D158, C:V159, C:A160, C:V161, C:Q162, C:L163, C:E164, C:G181, C:A182, C:F183, C:A185, C:S186, C:H189, C:C190, C:L192, C:P193, C:Q194, C:L195, C:S196, C:S197, C:P198, C:R199, C:L200, C:A201, C:V202, C:R203	STAAFHISSLLEKMTSSDKDFRFMATSDLMSELQKDSIQLDEDSERKVVKMLLRLLEDKNGEVQNLAVKCLGPLV**A**KVKEYQVETIVDTLCTNMRSDQLRDIAGIGLVGLATNVCRKITGQLTSAIAQQEDVAVQLEGAFASHCLPQLSSPRLAVR	0.5092 (Probable ANTIGEN).	Probable NON-ALLERGEN	IgG Epitope (1.117 *)	0.815
4LG1/A	A:D11, A:P12, A:L13, A:S15, A:F16, A:V17, A:R29, A:Q31, A:Q32, A:T55, A:P56, A:E57, A:S59, A:G60, A:D61, A:G62, **A:D63**, A:H64, A:A65, A:L66, A:S67, A:R68, A:R69, A:G89, A:A90, A:M106, A:N109, A:M110, A:K112, A:H113, A:L114, A:V115, A:T116, A:G117, A:D138, A:K200, A:I201, A:P202, A:L203, A:E204, A:K205, A:D207, A:E208, A:E209, A:Y210, A:R211	DPLSFVRQQTPESGDG**D**HALSRRGAMNMKHLVTGD KIPLEKDEEYR	0.6120 (Probable ANTIGEN).	Probable NON-ALLERGEN	IgG Epitope (0.999 *)	0.669
1O7A/A	A:P60, A:L61, **A:L62**, A:V63, A:K64, A:M65, A:T66, A:P67, A:N68, A:L69, A:L70, A:H71, A:L72, A:A73, A:P74, A:E75, A:N76, A:F77, A:Y78, A:I79, A:S80, A:H81, A:S82, A:P83, A:N84, A:S85, A:T86, A:A87, A:G88, A:P89, A:S90, A:C91, A:T92, A:E95, A:H102, A:G103, A:F106, A:G107, A:T122, A:Q123, A:V124, A:Q125, A:Q126, A:L127, A:L128, A:V129, A:S130, A:I131, A:T132, A:L133, A:Q134, A:S135, A:E136, A:C137, A:D138, A:A139, A:D146, A:L152, A:V153, A:K154, A:E155, A:P156, A:V157, A:A158, A:V159, A:K161, A:N163, A:Y180, A:Q181, A:D182, A:S183, A:Y184, A:G185, A:T186, A:F187, A:T188, A:I189, A:N190, A:E191, A:S192, A:T193	PL**L**VKMTPNLLHLAPENFYISHSPNSTAGPSCTEHGFGTQVQQLLVSITLQSECDADLVKEPVAVKNYQDSYGTFTINEST	0.4546 (Probable ANTIGEN).	Probable NON-ALLERGEN	IgG Epitope (1.035 *)	0.75

Neoepitopes were predicted and prioritized based on their binding affinity to MHC class I/II molecules, along with LBL/CBL epitopes identified using various immunoinformatics tools. *: Indicates statistically significant values as determined by the IgPred server.

### Neoepitopes antigenicity and allergenicity evaluation

Using VaxiJen (58) version 2.0, the neoepitopes were further investigated for their potential as antigens. The threshold value of 0.4 was considered. In order to circumvent the drawbacks of alignment-dependent sequence approaches, the VaxiJen categorized antigens using auto cross-covariance (ACC), a unique alignment-independent method that transforms protein sequences into uniform vectors of major amino acid characteristics (58). It is crucial to forecast the allergenicity of vaccine candidates. AllerTOP 2.0 (59) was used to calculate the proposed protein’s allergenicity. Using auto cross-covariance (ACC), which describes residue hydrophobicity, size, abundance, and helix- and β-strand-forming propensities, the AllerTOP approach predicts the allergenicity of recombinant proteins. ToxiPred (60) was used to assess toxicity. Non-toxic and “probable non-allergenic” peptides were chosen (**Table 1**, **Supplementary Data S3**).

### Molecular docking simulation of mouse MHC alleles-neoepitope and B-cell receptor (BCR)-neoepitope interactions

Molecular docking with the GalaxyPepDock web server (61) was used to assess the interaction performance between anticipated neoepitopes and their binding alleles. The frequent mouse alleles’ crystal structures, H-2-Kb (4PV9, Chain A), H-2-Db (7N9J, Chain A), I-Ab (4P23, Chain C), and BCR (8EMA, Chain C-D), were obtained from the Protein Data Bank (PDB) in PDB format to facilitate this analysis. The protein and ligand (neoepitopes) were separated from the complex structures using Discovery Studio v16.0.0.400 since the recovered crystal structures were in the complex form of the two substances. The hydrogen bonding pattern, similarity score, and accuracy score were used to determine which of the resulting peptide-protein complexes was the best. The Discovery Studio v16.0.0.400 tool was also employed to visualize the complex’s binding mode and binding interaction. In this section, two additional docking tools, HPEPDOCK and CABS, were also utilized for further confirmation of the docking results. HPEPDOCK is known for its efficient blind peptide–protein docking capabilities through a hierarchical algorithm that integrates available peptide binding information from the Protein Data Bank (PDB) (62). Meanwhile, CABS offers a coarse-grained approach that allows for efficient sampling of peptide conformations and their interactions with target proteins (63). Together, these tools provide a robust framework for validating the interaction predictions made by GalaxyPepDock, enhancing the reliability of the docking analysis performed in this study.

### Construction of MNEV and evaluate the physicochemical properties

Mouse LBL, CBL, HTL, and CTL epitopes derived from anticipated neoantigens were integrated into the creation of the MNEV. The linkers used to join these selected epitopes included Lys-Lys (KK) for LBL and CBL, Gly-Pro-Gly-Pro-Gly (GPGPG) for HTL, Ala-Asp (AD) linker, and Ala-Arg-Tyr (ARY) spacer for CTL epitopes (64, 65). The strategic use of these linkers aimed to ensure that the vaccine’s immunogenic properties were preserved. The 50S ribosomal protein L7/L12 (Accession No.: P9WHE3) was chosen as an adjuvant to increase the vaccine candidate’s immunogenicity (65, 66). A flexible EAAAK linker was used to bind this protein to the chimeric sequences’ N-terminus and the mouse Igκ signal sequence (MGMQVQIQSLFLLLLWVPGSRG) for vaccine secretion designed was also placed at the N end of the structure. To aid in purification by affinity chromatography, a 6-histidine tag was added to the construct’s C-terminus in addition to its primary constituents. These components work together to guarantee that all of the chosen epitopes are efficiently connected, enabling proper folding and functioning while preserving high immunogenicity and antigenicity.

An efficacious vaccine requires not only the capacity to elicit a robust immune response but also the possession of appropriate biochemical attributes. To evaluate the physicochemical properties of vaccines, we employed ProtParam (https://web.expasy.org/protparam/), a web-based tool that calculates parameters such as the aliphatic index, theoretical isoelectric point (pI), molecular weight, predicted half-life, amino acid composition, instability index, and grand average of hydropathicity (GRAVY). The aliphatic index serves as an indicator of a protein’s thermal stability. At the pI—defined as the pH at which a molecule exhibits no net surface charge—proteins display minimal solubility and maximal instability due to the absence of repulsive charges. The half-life represents the duration required for half of the synthesized protein to be degraded or cleared from the cellular environment. Proteins are expected to be stable if their instability index is less than 40 (67). The developed vaccine is hydrophilic if its GRAVY value is negative, and hydrophobic if its value is positive (68).

### Secondary and tertiary structure prediction and refinement

We used two well-known bioinformatics tools, PSIPRED)v3.3 web server( (69) and the Prabi server (67), to predict the secondary structure of the vaccine design. The Prabi server is a well-known tool for secondary structure prediction that analyzes amino acid sequences using default window width and similarity threshold settings. In addition to Prabi, we utilized PSIPRED, which enhances prediction accuracy by leveraging PSI-BLAST results through two feed-forward neural networks. This method allows for a more nuanced understanding of the protein’s secondary structure by incorporating evolutionary information from homologous sequences.

Applying the concept of sequence-to-structure-to-function, the publicly available online server I-TASSER (https://zhanglab.ccmb.med.umich.edu/I-TASSER/) was used to estimate the tertiary structure of the MNEV construct. The I-TASSER server was rated as the best server for protein structure prediction in the previous five community-wide CASP studies (70, 71). Using amino acid sequences, the I-TASSER service automatically produced excellent tertiary structure models of protein molecules. Here, we assessed the prediction model quality using the confidence score (C score). The model’s believability increases with a greater C score, which falls between -5 and 2. Some error-sensitive root mean-square deviation (RMSD) concerns were addressed using the template modeling (TM) score. Therefore, it was necessary to use refinement techniques to increase the anticipated model’s precision. The GalaxyRefine server (http://galaxy.seoklab.org/cgi203bin/submit.cgi?type=REFINE) was used to refine the predicted model by repacking side chains and applying molecular dynamics to relax the overall structure (72). The ProSA-web server (https://prosa.services.came.sbg.ac.at/prosa.php) and the SAVES v6.0 server (https://saves.mbi.ucla.edu/) were used to validate the model. The structural integrity was further validated through Ramachandran plot analysis using RAMPAGE, followed by assessments with ProSA-web and the ERRAT server to evaluate the accuracy of the structure.

### Prediction of cleavage sites

Protease processing was predicted using NetChop 3.1 (http://tools.iedb.org/netchop/) (Threshold 0.5) (73). Additionally, Proteasomal cleavage and TAP transport were predicted using the IEDB processing tool (http://tools.iedb.org/processing/).

### Molecular docking analysis of the optimized MNEV construct with TLR3, TLR4, and TLR9

The Cluspro docking server (https://cluspro.bu.edu/login.php) was used to identify the interface between the vaccine construct and TLR3, TLR4, and TLR9 (74). The server generated ten docking solutions, with the lowest-energy cluster selected for analysis due to its high binding affinity. Additionally, the Discovery Studio visualizer v16.0.0.400 tool was used to graphically depict the various residues that interfered with TLR3, TLR4, and TLR9 and the vaccine design (75).

### MM-GBSA binding free energy

The molecular mechanics/generalized born surface area (MM-GBSA) analysis was carried out in conjunction with the docking, keeping all of the default settings (76–78).

### *In silico* cloning and optimization of designed MNEV

The MNEV construct’s codons were optimized using the Java Codon Adaptation Tool (JCat). The proportion of GC content produced in the JCat output and the Codon Adaptation Index (CAI) were used to evaluate the amount of protein expression. The SnapGene tool was then used to clone the vaccine’s optimized sequence into the Plenti-Giii-Cmv-Gfp-2A-Puro vector. For both codon optimization and *in silico* cloning in eukaryotic cells, default settings were used. A His6 tag was added to the end of the sequences to aid in purification. BamHI and XbaI restriction sites were added to flank the 5′ and 3′ ends of the DNA sequence, respectively.

### *In silico* immune simulation

To evaluate the immune response elicited by the MNEV, C-ImmSim (http://150.146.2.1/C-IMMSIM/index.php), an online simulation server, was utilized (79). The server integrates machine learning to simulate immune dynamics. In the mammalian immune system, it predicted both humoral and cellular immunity (79, 80). Three doses of the anticipated vaccine design spaced four weeks apart were administered using the server under the default settings, with simulation volume 50 and simulation steps 1000.

### *In vivo* studies

#### Animal care and BM-MSCs isolation

To evaluate potential immune responses of the designed vaccine, immune simulation was performed *in vitro* and *in vivo*. Female C57BL/6 mice, each weighing approximately 20 ± 1 g, were obtained from the Pasteur Institute in Tehran, Iran. All mice were housed in accordance with the regulations of the National Institute of Genetic Engineering and Biotechnology (NIGEB). Throughout the duration of the study, the mice had unrestricted access to standard laboratory chow and water.

### Lentiviral vaccine

The designed MNEV construct was purchased (Shinegene, China), and subsequently cloned into the third-generation lentiviral transfer vector pLenti-GIII-CMV-GFP-2A-Puro (abm, Canada, LV053). For control purposes, lentiviral particles lacking the vaccine construct were utilized as the empty lentiviral vector (LV) control. The production of LVs was conducted following established protocols (81). To concentrate the LV, polyethylene glycol 8000 (PEG8000) was employed, and the concentrated virus was resuspended in endotoxin-free phosphate-buffered saline (PBS) (Sigma). The viral titer was determined using flow cytometry on HEK293T cells, and the resulting viral particles were stored at -80°C for future vaccination applications.

### Expression of MNEV by mesenchymal stem cells

Mouse bone marrow-derived mesenchymal stem cells (mBM-MSCs) were isolated and characterized from 8-week-old C57BL/6 mice, following previously established protocols (81). The mBM-MSCs were subsequently transduced with MNEV-LV or empty LV. The expression of MNEV was assessed using reverse transcription polymerase chain reaction (RT-PCR). To confirm protein expression and secretion into the conditioned media (CM), a His-tag ELISA kit (GenScript, L00436) was employed, adhering to the manufacturer’s guidelines. As controls, CM from MSCs transduced with empty LV or CM from untransduced MSCs were utilized.

### Mice immunization

Mice were acclimated for a minimum of two weeks prior to the commencement of the study, after which they were randomly assigned to one of three groups (n = 18). The first group, designated as the Mock group, received a subcutaneous injection of 100 μL of PBS and served as a negative control (n = 6). The second group, referred to as the Empty LV group, was administered a subcutaneous injection of empty LV particles (n = 6). The third group, known as the MNEV group, received a subcutaneous injection of 100 μL of LVs expressing the MNEV (MNEV-LV) on the right flank, with a dosage of 2 × 10^7 infectious units (I.U) in 100 μL of PBS (n = 6). Immunizations were administered on days 0, 14, and 28, with a two-week interval between each of the three injections.

### Serum IgG detection by ELISA

Blood was collected from the immunized mice through tail bleeding two weeks after the final injection, and the total serum IgG measured using Mouse Total IgG Uncoated ELISA Kit (Invitrogen, USA).

### IFN-γ production

Mice from each group were euthanized using an overdose of ketamine and xylazine, and spleens were aseptically harvested from immunized or control groups two weeks after the final injection. They were then mechanically homogenized into single-cell suspensions and treated with RBC lysis buffer to lyse red blood cells. Splenocytes were isolated and cultured in 24-well plates using RPMI 1640 medium (Sigma, USA) supplemented with 10% fetal bovine serum (FBS) (Sigma, USA) and 1% antibiotic mixture containing penicillin and streptomycin (Sigma, USA). The splenocytes were re-stimulated with MSC-MNEV-CM and incubated at 37°C in a humidified atmosphere with 5% CO2 for 48 hours. Following incubation, the levels of IFN-γ cytokine in the supernatants were measured using the IFN-γ Mouse Uncoated ELISA Kit (Invitrogen, USA) according to the manufacturer’s instructions.

### Flow cytometric evaluation of lymphocyte cells population

For a detailed evaluation of T-cell subsets and total B cells in response to the immunization and subsequent re-stimulation, following the immunization of mice, the cultured splenocytes from each group were restimulated with MSC-MNEV-CM. After stimulation, the splenocytes were analyzed using flow cytometry. CD3+ T cells were first identified and gated, and the proportions of CD4+ and CD8+ T cells within the CD3+ population were then assessed. The staining process employed FITC-conjugated anti-CD4, APC-conjugated anti-CD8, and PE-labeled CD3 antibodies. To assess total B cell levels in splenocytes from mice in each study group via flow cytometry, cells were labeled with FITC-conjugated CD19 antibodies. Subsequent analysis was conducted using a BD FACSCalibur flow cytometer.

### Granzyme B activity assay

Cytolytic activity was evaluated by quantifying Granzyme B (GrB) protein levels in the supernatants of MNEV-stimulated splenocytes from three mice per group. These measurements were conducted two weeks following the final administration, adhering to the protocol provided with the Mouse Granzyme B ELISA Kit (Invitrogen, USA). All assays were performed in triplicate for each mouse to ensure reliability and consistency.

### Statistical analysis

Comparisons between immunization groups were conducted using one-way ANOVA, supplemented by Tukey’s *post hoc* tests to identify significant pairwise differences. A p-value less than 0.05 was considered statistically significant; specific levels of significance were denoted as follows: * (p ≤ 0.05 but > 0.01), ** (p ≤ 0.01 but > 0.001), and *** (p < 0.001).

## Result

### Identification of mutation-associated neoantigens in LL/2 and A549 tumors

**Figure 1:**
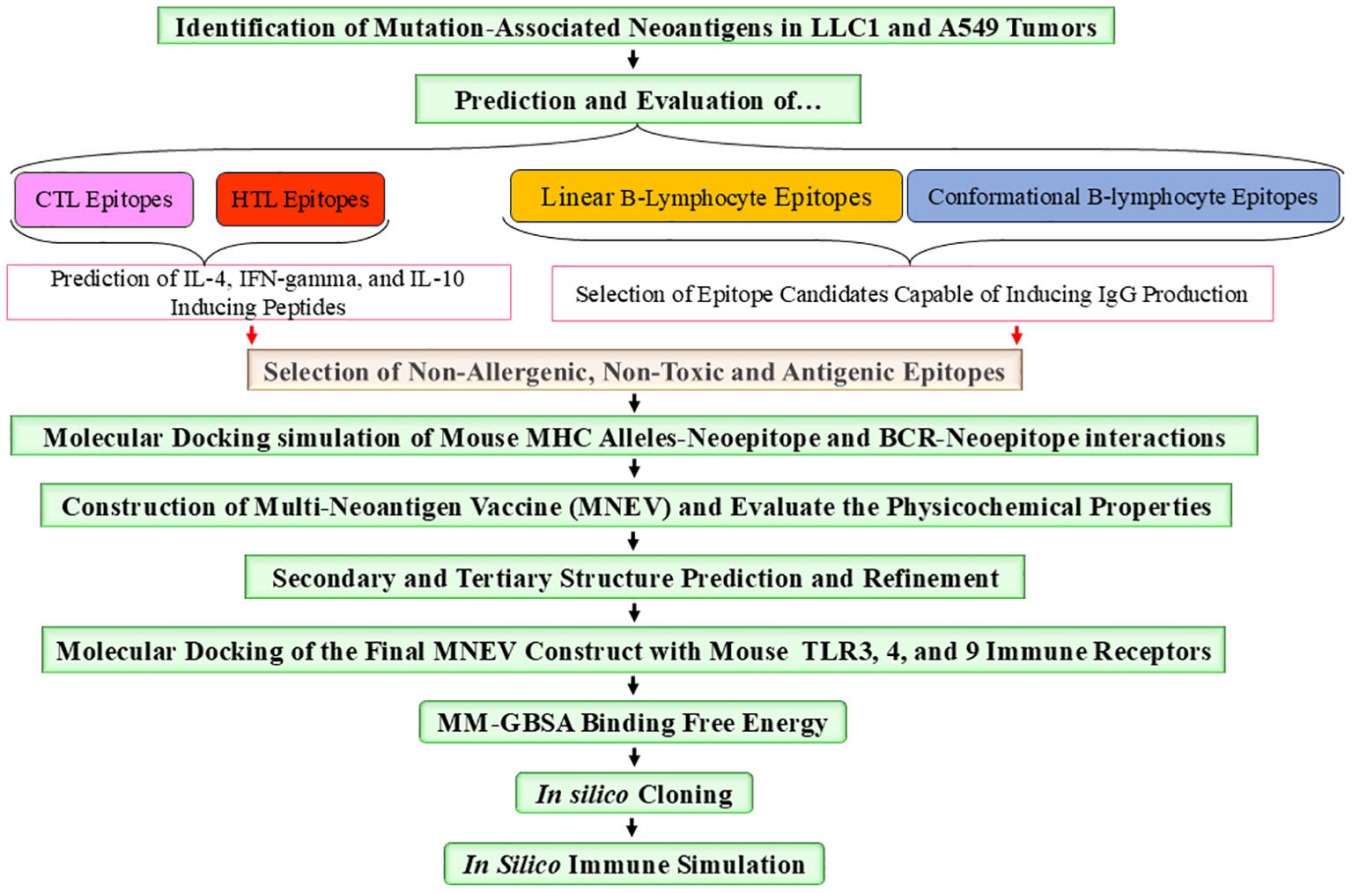
An overview of the workflow of the *in silico* study.

**Figure 2:**
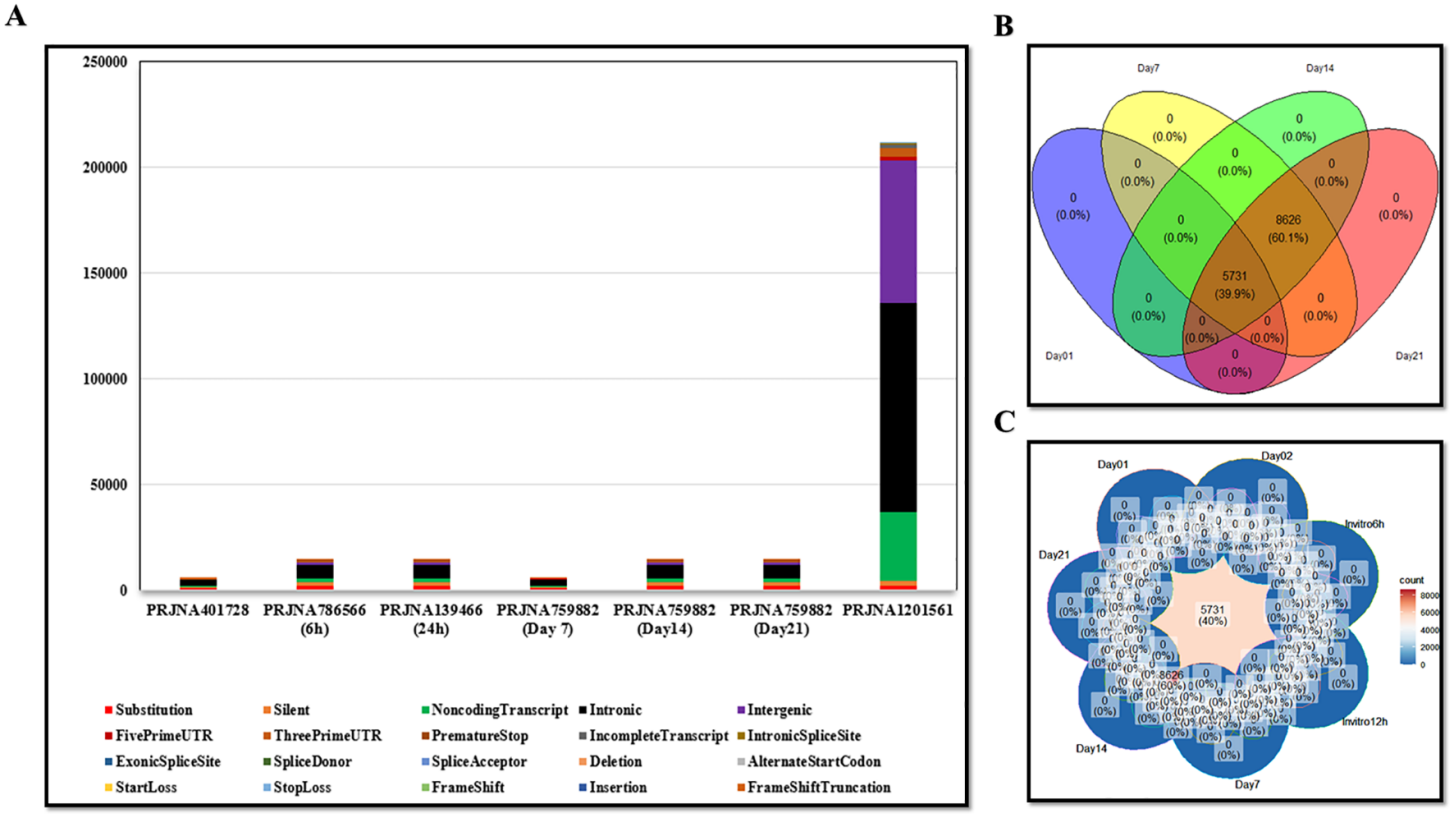
**(A)** Graph showing the types of mutations observed in all analyzed datasets, including both mouse and human samples. **(B, C)** Venn diagrams illustrating the distribution of substitution mutations, indicating that 40% of the LL/2 substitution mutations are shared across seven datasets as well as in the overall analyzed data. The show emphasizes the repeatability of the mutations observed in all the reviewed data.

The tumor and normal DNA samples from Whole Genome Sequencing data were aligned against the Mouse GRCm38/mm10 and GRCh38/hg38 reference genome using BWA-MEM, while the tumors’ RNA was aligned using STAR. Somatic variant calling was performed using MuTect, and the results were aggregated by taking the union of the identified variants. The VCF files are included in the supplementary data (**Supplementary Data S1**). The results were annotated with the help of VEP, and various types of mutations were observed, including synonymous, missense, stop gained, frameshift variant, stop lost, splice region variant, start lost, NMD transcript variant, and stop retained (The annotated results were included in the **Supplementary Data S1**). Multiple software packages are available to predict the protein-level effects of coding mutations. Predicting the impact of a DNA mutation on protein function is incomplete without considering the specific transcripts in which it occurs. Somatic mutations can be associated with selective splicing of particular RNA isoforms and may co-occur with other genomic variants. To address these complexities, tumor RNA sequencing data were used to determine the mutant coding sequences. Due to sequencing errors, splicing diversity, and tumor heterogeneity, multiple coding sequences can be inferred for each mutation from supporting RNA reads. To resolve these challenges, we utilized the isovar tool (available at https://github.com/hammerlab/isovar), which leverages RNA data to assemble the most abundant coding sequence for each mutation (see **Supplementary Data S2**). Initially, the total exome sequencing of LL/2 cells revealed 962 missense mutations specific to this cell line. Furthermore, analysis across all datasets indicated the presence of 2003 missense mutations in the A549 cell line (**Supplementary Data S4**). **Figure 1** provides an overview of the MNEV design workflow. The types of mutations observed across all analyzed data are summarized in the **Supplementary Data S4**. A Venn diagram, illustrating LL/2 substitution mutations, was created for all seven datasets surveyed, revealing that 40% of substitution mutations are recurrent and common across all datasets (**Figure 2**).

### Epitope prediction and designing of the MNEV

**Table 1**, **Supplementary Data S3** list epitopes with the highest predicted binding affinities for activating T cells and B cells. Further analysis identified protein domains most likely to contain immunogenic epitopes. The final MNEV was then designed and constructed utilizing linkers. The use of linkers in the MNEV construct for lung cancer is strategically important for ensuring proper epitope presentation and enhancing immune responses and the appropriate linkers are necessary to maintain the functional 3D structure of selected epitopes. Specifically, Lys-Lys (KK) linkers were employed for linking B-cell epitopes, while Gly-Pro-Gly-Pro-Gly (GPGPG) linkers were used for connecting HTL epitopes. For CTL Ala-Asp (AD) linker and Ala-Arg-Tyr (ARY) spacer (ADARY) were utilized. The choice of these linkers is based on their ability to facilitate the appropriate spatial arrangement of the epitopes, which is crucial for effective immune recognition. The KK linker promotes the formation of stable interactions with B-cell receptors, enhancing the activation of B cells and subsequent antibody production. The KK linker serves as a target sequence for the lysosomal protease cathepsin B, a key enzyme involved in antigen processing for MHC II antigen presentation. By linking two peptides via the KK linker, each peptide can be exposed to antibodies while preventing the formation of antibodies against the novel amino acid sequence created by the fusion of the two peptides. The flexible connecting peptide (GPGPG linker) serves to prevent the formation of junctional epitopes and aids in immune processing, ensuring that the HTL epitopes are effectively presented to T cells. The remaining linkers were used mainly considering their ability to induce HTL immune response (GPGPG). Meanwhile, the AD linker and ARY spacer are designed to optimize the interaction between CTL and HTL epitopes, thereby promoting a robust cellular immune response. To optimize peptide proteasomal cleavage for HLA-I presentation, specific amino acid sequences such as ARY, RY, GR, or TV were utilized as linkers or spacers when necessary. The 50S ribosomal protein L7/L12 (Accession no. P9WHE3, Locus RL7_MYCTU) was incorporated as an adjuvant at the N-terminal ends using the EAAAK linker—a rigid linker that adopts an alpha helix structure. This linker serves as a spacer to enhance vaccine stability and maintain optimal inter-domain distances, thereby facilitating independent domain function and augmenting the immunogenicity of the vaccine construct. By carefully selecting these linkers, we aim to maximize the immunogenicity of the MNEV, ultimately improving its efficacy against lung cancer.

### Identification and selection of T-cell Neoepitopes

By stimulating CTLs and boosting immune responses through the generation of cytokines, HTLs, especially CD4+ T cells, are essential in the destruction of cancer cells. Through processes like granzyme B and perforin release, they can directly destroy tumor cells, and their IFN-γ production is essential for fostering anti-tumor immunity and preventing metastasis. According to recent research, HTLs have a variety of roles in cancer immunotherapy, including boosting other immune cells and directly attacking tumor cells (82). Determining if epitopes have the potential to be immune protective is the first step in using bioinformatics to build multi-neoepitope subunit vaccines. MHC displays T-cell epitopes in a linear format of 9–20 amino acids. This information makes it easier to accurately simulate the interaction between ligands and T-cells. The most selective stage in presenting an antigenic peptide to the T-cell receptor (TCR) is the binding of the MHC molecule. When antigen is presented on the surface of antigen-presenting cells (APCs), T cells’ surface T cell receptors (TCRs) connect to MHC molecules, allowing T cells to identify antigen. MHC I and MHC II molecules present T cell epitopes, which are recognized by two different T cell subsets, CD8+ and CD4+ T cells, respectively. As a result, CD8+ and CD4+ T cell epitopes need to be examined separately when predicting T cell epitopes. Using NetMHCPan 4.1 (BA), SYFPEITHI, NetMHCcons, NetCTLpan, five CTL epitopes were predicted for LL/2 and eight CTL epitopes were predicted for A549. The VaxiJen v2.0, ToxiPred, and AllerTOP v. 2.0 servers were used to assess the antigenicity, toxicity, and allergenicity of these epitopes, respectively. All selected antigen epitopes were non-allergenic and non-toxic.

*In silico* analysis was performed to predict MHCII-binding epitopes in LL/2 and A549 cell lines. For the LL/2 cell category, four HTL epitopes were identified based on predictions from Rankpep (top 2%), NetMHCIIpan-4.0 BA (IC50 < 500nM, Percentile Rank ≤ 10), IEDB (percentile > 0.10), MHC2pred (score > 0.2), and NetMHCII 1.1 (SMM-Align, Adjusted Rank < 10) algorithms. In the A549 cell category, we predicted 48 neoepitopes for MHCII using NetMHCIIpan-4.1 BA (IC50 < 500nM, Percentile Rank ≤ 10), NetMHCII 1.1 (SMM-Align, IC50 < 500nM, Adjusted Rank < 5), and CONSENSUS 2.2 (Adjusted Rank < 10) prediction servers. The identified epitopes demonstrated the ability to bind to specific MHC II alleles, including IAb in the LL/2 cell line and HLA-DRB104:04, HLA-DRB104:01, and HLA-DRB1*07:01 in the A549 cell line. Furthermore, we assessed their antigenicity, toxicity, and allergenicity. Notably, all selected antigenic epitopes were found to be non-allergenic and non-toxic (**Table 1**, **Supplementary Data S3**).

Through the production of important cytokines like IL-4, IL-10 and IFN-γ, the strategic design of multi-neoepitope anti-cancer vaccines employing neoantigenic epitopes shows promise in generating strong immune responses that these data are summarized in **Table 1** and further detailed in **Supplementary Table S3**. Optimizing vaccine formulations to achieve effective tumor suppression while maintaining patient safety will need an understanding of the interactions between these cytokines. Neoepitope-mediated cytokine secretion varied between cell lines. In LL/2 cells, we observed that three neoepitopes stimulated IL-4 secretion and two stimulated IL-10 secretion. In contrast, A549 cells exhibited a more robust response, with 28 neoepitopes inducing IL-4 secretion and all neoepitopes inducing IL-10 secretion. Stimulation of interferon-gamma (IFN-γ) secretion was observed for two neoepitopes in LL/2 and 22 in A549 cells. For vaccines to be effective, it is essential to comprehend how these cytokines interact: (1) Interleukin-4 (IL-4) is known to boost B cell activation and Th2 responses, which in turn increases the generation of antibodies. Interestingly, under some circumstances, IL-4 can also promote the production of IFN-γ. Studies reveal that IL-4 mainly triggers the production of IFN-γ via natural killer (NK) and NKT cells instead of traditional T cells, indicating a complicated interaction between these cytokines, (2) Interferon-gamma (IFN-γ) promotes antigen presentation through MHC II molecules and increases macrophage activation, both of which are essential for mediating anti-tumor immunity. It has been demonstrated to reverse the effects of IL-4 on B cells and is mostly generated by Th1 cells, suggesting a regulatory balance between these two cytokines (**Table 1**, **Supplementary Data S3**).

### Prediction and assessment of LBL and conformational neoepitopes

A section of an antigen that is recognized by a specific BCR or, later, the antibody that is generated in a humoral reaction is known as a B-cell epitope. Based on its spatial shape, a B-cell epitope can be classified as either a conformational epitope or a linear epitope. While a conformational epitope, also referred to as a discontinuous epitope, is made up of sequential segments that are brought together in spatial proximity when the relevant antigen is folded, a liner epitope, also known as a continuous epitope, is made up of residues that are sequentially successive. Over 90% of B-cell epitopes are discontinuous, according to certain reports. Epitope prediction’s ultimate objective is to assist in the creation of compounds that can replicate the structure and functionality of a real epitope and take its place in vaccine development, medical diagnostics, and therapies. Utilizing ABCPred (threshold = 0.51, window length = 16) and BepiPred-2.0 (threshold = 0.35), we predicted linear B-cell epitopes in both cell lines. This analysis identified four potential epitopes (15–16 amino acids) in LL/2 cells and seven in A549 cells. The ElliPro server predicted four discontinuous B-cell epitopes among the neoepitopes of the LL/2 cell line, for which IgG antibodies could be produced. Five structural B-cell neoepitopes were predicted in the A549 cell line (minimum score = 0.5; maximum distance = 6 Å). A total of five structural B-cell neoepitopes were identified in each cell line. In LL/2 cells, the predicted scores ranged from a minimum of 0.693 to a maximum of 0.784. In A549 cells, the minimum and maximum predicted scores were 0.669 and 0.815, respectively. This work emphasizes how important both linear and conformational epitopes are for triggering immune responses, especially when it comes to developing vaccines and using them therapeutically. Designing more successful immunotherapy techniques will be aided by an understanding of these systems (**Table 1**; **Supplementary Data S3**).

### Neo-epitopes antigenicity and allergenicity evaluation

The AllerTOP tool was used to evaluate the allergenicity of neo-epitopes, while VaxiJen v2.0 (Threshold > 0.4) and Toxipred were used to evaluate their antigenicity and toxicity, respectively. All of the chosen neoepitopes were determined to be antigenic, non-toxic, and non-allergenic.

### Molecular docking simulation of the mouse MHC alleles-neoepitope and BCR- neoepitope interaction

**Figure 3:**
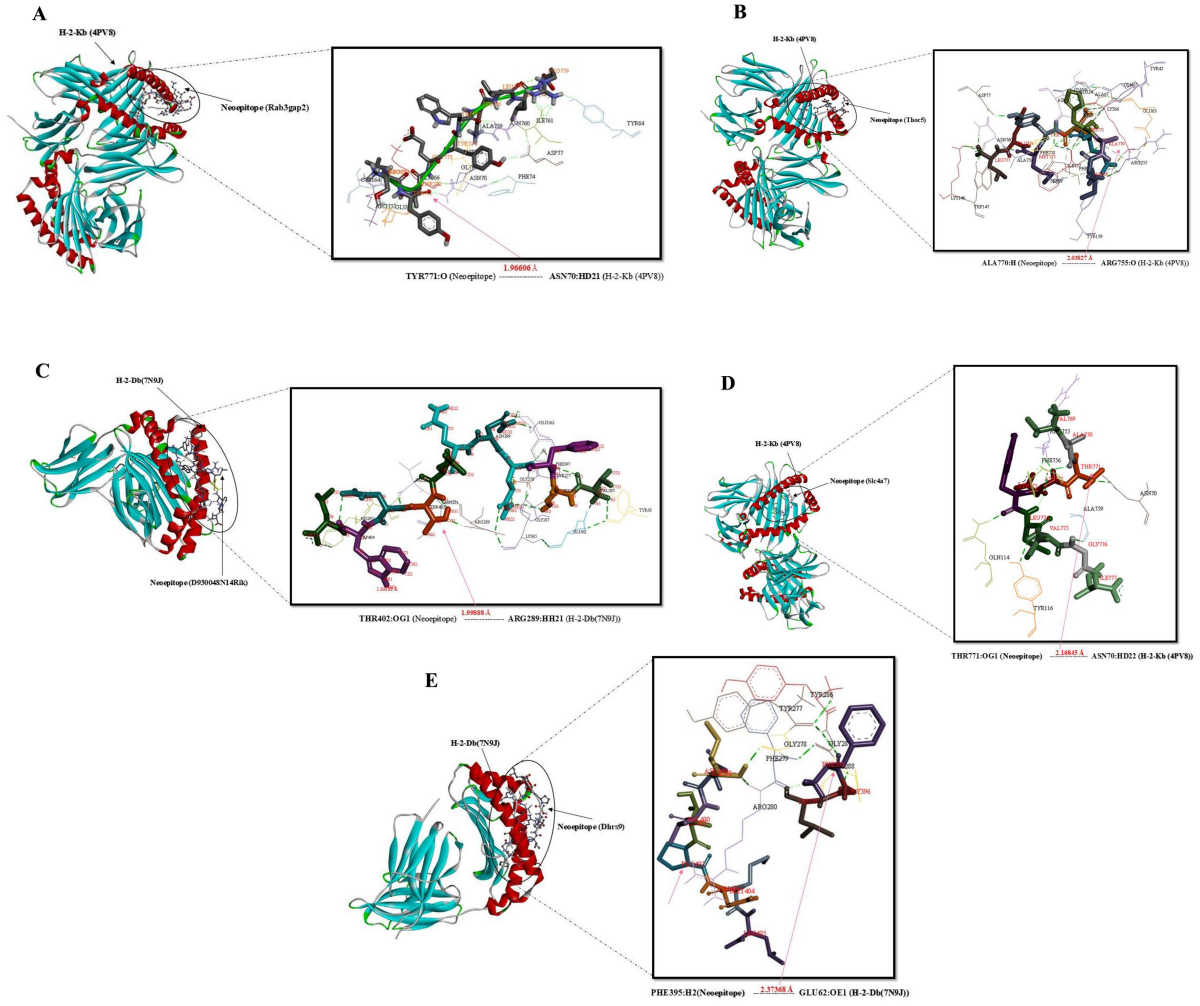
Docking positions of predicted peptide epitopes on 3D model of MHCI. This figure illustrates the docking positions of predicted peptide epitopes on the 3D models of Mouse MHCI molecules, specifically H-2-Kb (4PV9, Chain A) and H-2-Db (7N9J, Chain A), utilizing the GalaxyPepDock server. The connections between the neoepitopes and mouse MHC I alleles are depicted. The hydrogen bond distances between the mutated amino acids and the respective MHCI alleles are shown for each peptide. The peptides analyzed include: **(A)** SLYTEYWKLLR, **(B)** IAHEDYMEL, **(C)** VSFQNQLTNWL, **(D)** VATDYLVGI, **(E)** FGLINVTPNML.

**Figure 4:**
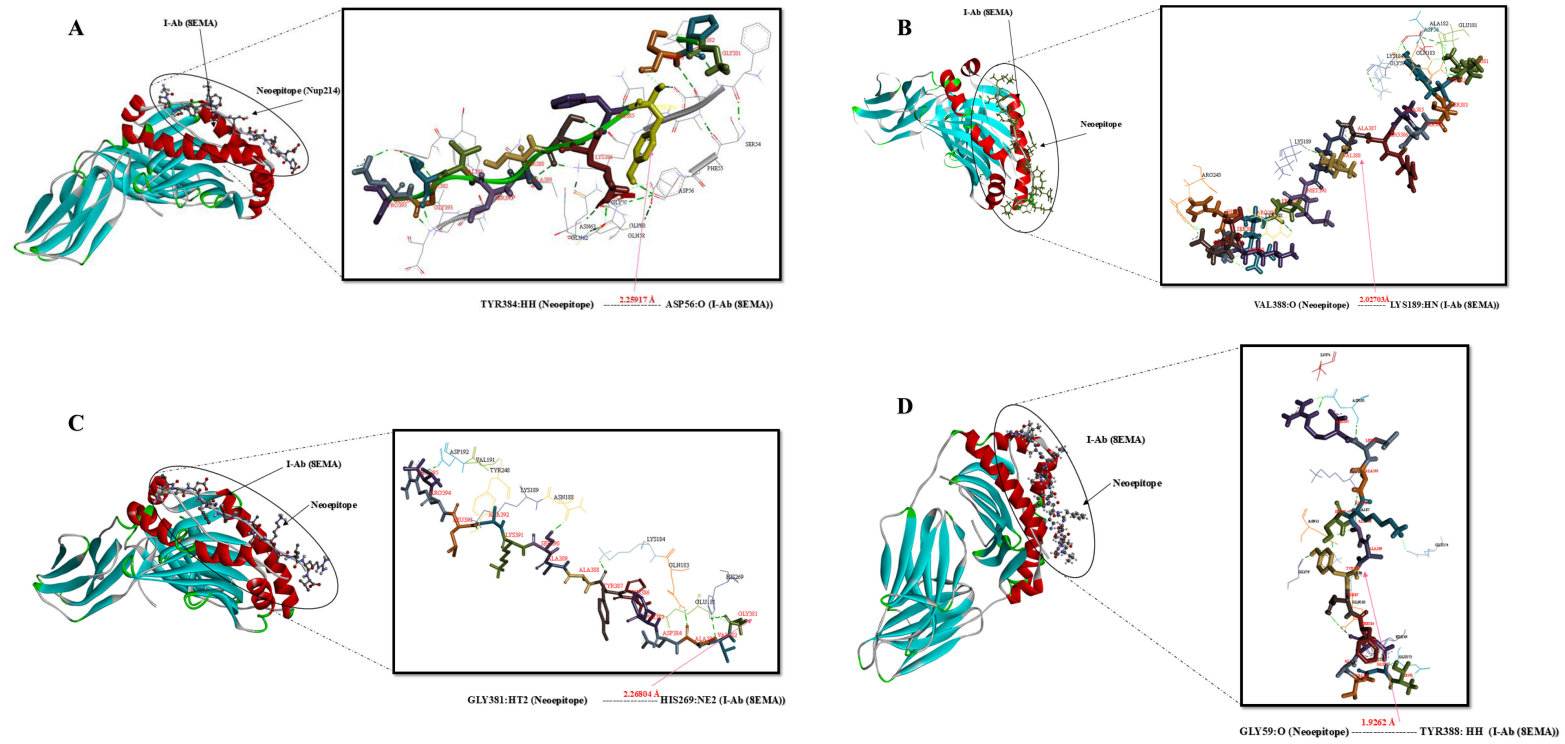
Docking positions of predicted peptide epitopes on 3D model of MHCII. This figure presents the docking positions of predicted peptide epitopes on the 3D model of the MHCII I-Ab (4P23, Chain C), utilizing the GalaxyPepDock server. The binding and interaction analyses of the peptides are displayed for the following sequences: **(A)** GPSYFKSSASVTGEP, **(B)** KYSSARAVRMPRHEKSP, **(C)** GVADFHYAASKALRV, **(D)** TGVADFHYAASKALR. The hydrogen bond distances between the mutated amino acids and their respective MHC II alleles are indicated for each peptide.

**Figure 5:**
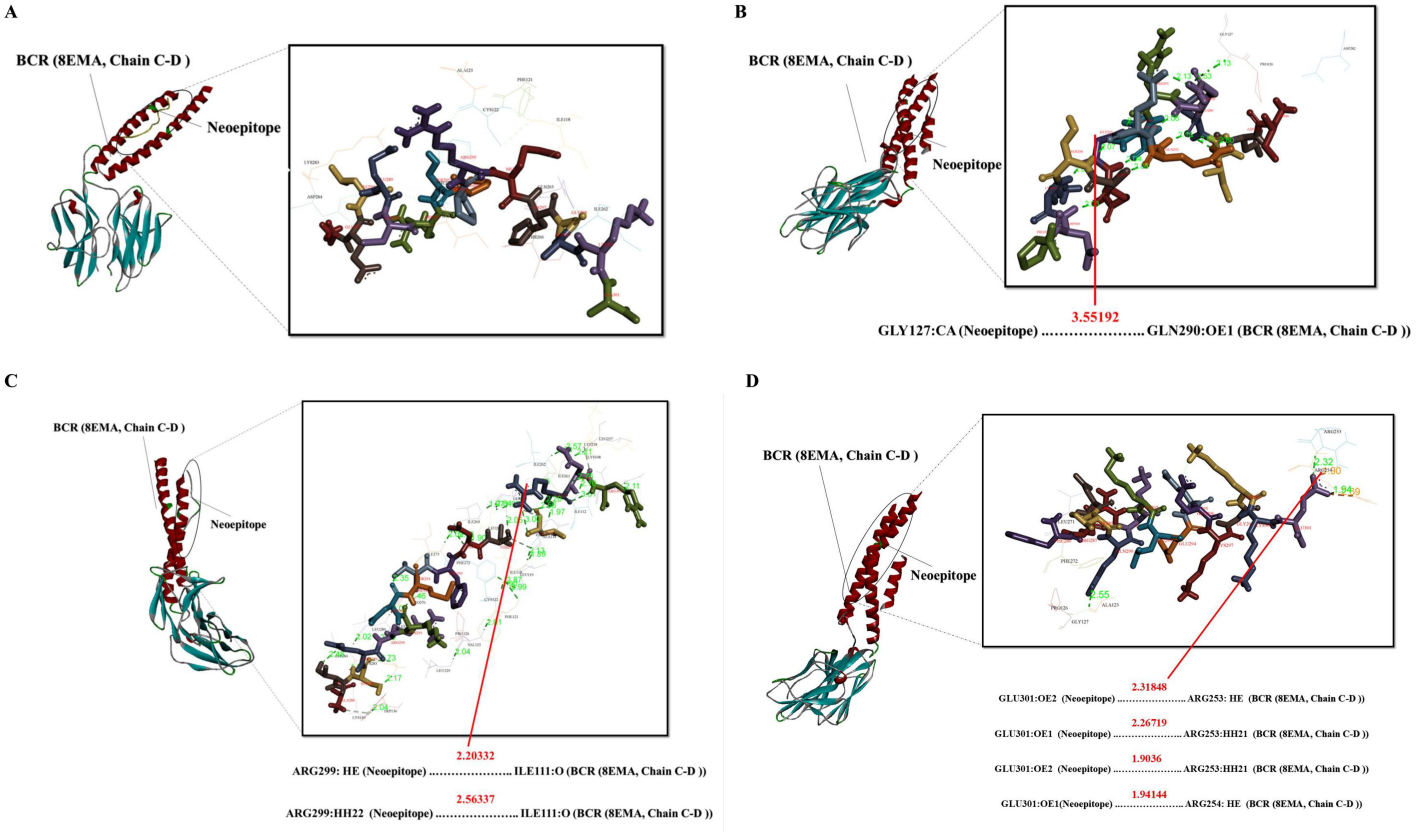
Docking positions of predicted peptide epitopes on the 3D model of BCR. This figure illustrates the docking positions of predicted peptide epitopes on the 3D model of the BCR, specifically 8EMA (Chain C-D), utilizing the GalaxyPepDock server. The binding and interaction analyses of the peptides are presented for the following sequences: **(A)** GELECRSPPRMHGAKA, **(B)** IDILQRRQEGQASKDP, **(C)** ATSERKDMTFDTLRNR, **(D)** FRDTQKKLEEEKGKKE. The hydrogen bond distances between the mutated amino acids and their respective BCR allele is indicated for each peptide.

A number of interaction metrics, such as hydrogen bonding patterns, similarity scores, and accuracy scores, were used to assess and rank the peptide-protein complexes. The chosen complexes’ binding modalities were examined using Discovery Studio visualizer v16.0.0.400 tool. In order to determine the structural basis, structural complexes of 4PV9, Chain A (H-2-Kb)/7N9J, Chain A (H-2-Db)/4P23, Chain C (I-Ab)/8EMA, Chain C-D (BCR) with neoepitopes were analyzed using a computational peptide-protein docking. **Figures 3**–**5** displays the interacting residues in each docked complex. These residues show that the neoepitopes and receptor proteins interact as much as possible. A thorough grasp of the interactions between peptides and proteins was made possible by this methodical approach, which also made it easier to identify the most intriguing complexes for more research. In relation to GalaxyPepDock, a TM score higher than 0.6 and an estimated accuracy greater than 0.8 indicate a strong interaction between the peptide and the allele. These values suggest that the docking results reflect a reliable and favorable binding conformation, supporting the potential efficacy of the peptide in eliciting an immune response (**Supplementary Data S4**). Lastly, the complex chosen by GalexyPepDock once more used the HPEPDOCK and CABS flexible connection server to conduct interaction similarity scores from chosen CTL, HTL neoepitopes. In the context of CABS-dock, a cluster density greater than 25 and approaching 135 may indicate a high quality of the docking results and the stability of the interactions. This range suggests that the models generated during the docking process are well-represented and exhibit significant similarity, reflecting reliable binding conformations between the peptide and the target protein. In the context of HPEPDOCK, a docking score below -150 kcal/mol is indicative of a strong binding affinity between the peptide and the target protein. Such a high score suggests that the peptide is likely to interact favorably with the target, which is essential for the development of effective therapeutic agents. Typically, docking scores in HPEPDOCK are expected to be negative and lower values (more negative) are generally preferred. This reinforces the peptide’s potential as a candidate for further experimental validation and development in therapeutic applications. The outcomes are shown in **Supplementary Data S4**. Also, hydrogenated links are the result of the neoepitopes connection and the MHCs/BCR alleles shown in **Supplementary Data S4**.

### Construction of MNEV and evaluation of its physicochemical properties

Using a variety of parameters, we carefully evaluated a few chosen neoepitopes before incorporating them into a specially designed MNEV framework. CTL, HTL, and B cell epitopes were connected using different linkers: KK linkers for B cell epitopes, GPGPG linkers for HTL epitopes, and ADARY linkers for CTL epitopes. Additionally, we used an EAAAK linker to carefully attach an adjuvant known as 50S ribosomal protein L7/L12 (Locus RL7_MYCTU) to the N-terminus (**Supplementary Data S4**). There are 472 amino acids in the vaccine. The generated vaccine demonstrated a high degree of antigenicity and safety with a noteworthy antigenicity score of 0.838232 (Probable ANTIGEN), a non-allergenic, and an allergenicity score of 0.6020. In order to confirm the new vaccine’s endurance, we also assessed its physicochemical properties. A detailed description of the attributes is provided in **Supplementary Data S4**. The ProtParam server was used to investigate the physiochemical characteristics of the MNEV design. This construct’s 472 amino acids have a molecular weight of around 51447.30 g/mol, a theoretical pI of 6.46, and a net charge of -2.6 at pH 7, indicating a slightly acidic nature. With an aliphatic index of 74.53 and an average half-life of 30 hours *in vivo* (in human reticulocytes), the construct is thermostable; in yeast and *Escherichia coli* (*E. coli*), it is >20 & >10 hours, respectively. According to reports, its GRAVY score was -0.546.

### Secondary and tertiary structure prediction and refinement

The functioning of the protein sequences is closely linked to the secondary structural characteristics. The most prevalent secondary structures in proteins are the α-helix, β-sheet, β-turn, and random coil. Protein secondary structure describes how the polypeptide chain of a protein folds and wraps. Two servers were used to evaluate the secondary structural elements of the vaccine, such as random coils, alpha helices, and beta turns. The predictions from PSIPRED corroborated those from Prabi, confirming that the vaccine’s secondary structure is predominantly composed of alpha helices, with a significant amount of random coil content (**Supplementary Data S4**).

The I-TASSER server was used to predict the 3D structure of the MNEV construct. The server generated five 3D models, and the highest-quality model was selected based on confidence score (C-score) and other quality metrics. C-scores, typically ranging from -5 to 2, indicate model confidence, with higher scores reflecting greater reliability. The QMEAN results validated the greater quality of the chosen model. The peptide’s tertiary structure was predicted using the I-TASSER service. The prediction model demonstrated great reliability, as evidenced by its C score of -1.02, TM score, and RMSD of 0.59 ± 0.14 and 9.5 ± 4.6Å, respectively (**Supplementary Data S4**). ProSA-web provided the overall quality score for protein structures (**Supplementary Data S4**). The unrefined model’s Z-score was -5.26 (**Supplementary Data S4**), and following refining, it dropped to -5.74 (**Supplementary Data S4**). According to the ProSA result, the chosen model has to be improved because it did not show up in the range of natural proteins with comparable sizes. Energy was reduced and the chosen primary model was improved. The chosen 3D model is now of higher quality thanks to the model refining and energy minimization runs. The modeling process provided five potential structures, from which we selected the optimal model based on its overall quality score and Ramachandran plot analysis. For the prM protein, Model 4 was chosen due to its high ERRAT score of 100 and favorable structural characteristics, with 89.9% of residues located in the favored region, 7.4% in the allowed region, and 2.2% in the disallowed region. The selected model was visualized using Chimera software.

### Prediction of cleavage sites

Enzymes within the proteasome complex play a critical role in the degradation of peptide bonds, converting proteins into smaller peptide fragments. Following proteasomal cleavage, these peptide molecules bind to MHCI molecules and are subsequently transported to the cell membrane, where they are presented to cytotoxic T cells. This transfer is facilitated by TAP which transports the peptides into the endoplasmic reticulum. The ability of our vaccine to generate peptides that can effectively bind to MHC I and stimulate cytotoxic T-cells is paramount for its efficacy. To investigate proteasomal cleavage, we utilized the NetCHOP, identifying a total of 172 immunoproteasomal cleavage sites. These findings suggest that our vaccination strategy may indeed activate cytotoxic T-cells (**Supplementary Data S4**). To further assess the immunogenic potential of the vaccine peptides, we employed advanced proteasomal cleavage prediction methodologies. The high proteasomal scores (Threshold =0.5) indicate that these peptides possess favorable characteristics for binding to MHC I molecules while also being likely to undergo effective cleavage by the proteasome. Previous studies have indicated that peptides with higher scores are more prone to cleavage at their termini rather than at internal sites, thereby enhancing their potential as T cell epitopes. This underscores the significance of our findings in the context of developing an effective immunotherapeutic approach.

### Molecular docking simulation results

**Figure 6:**
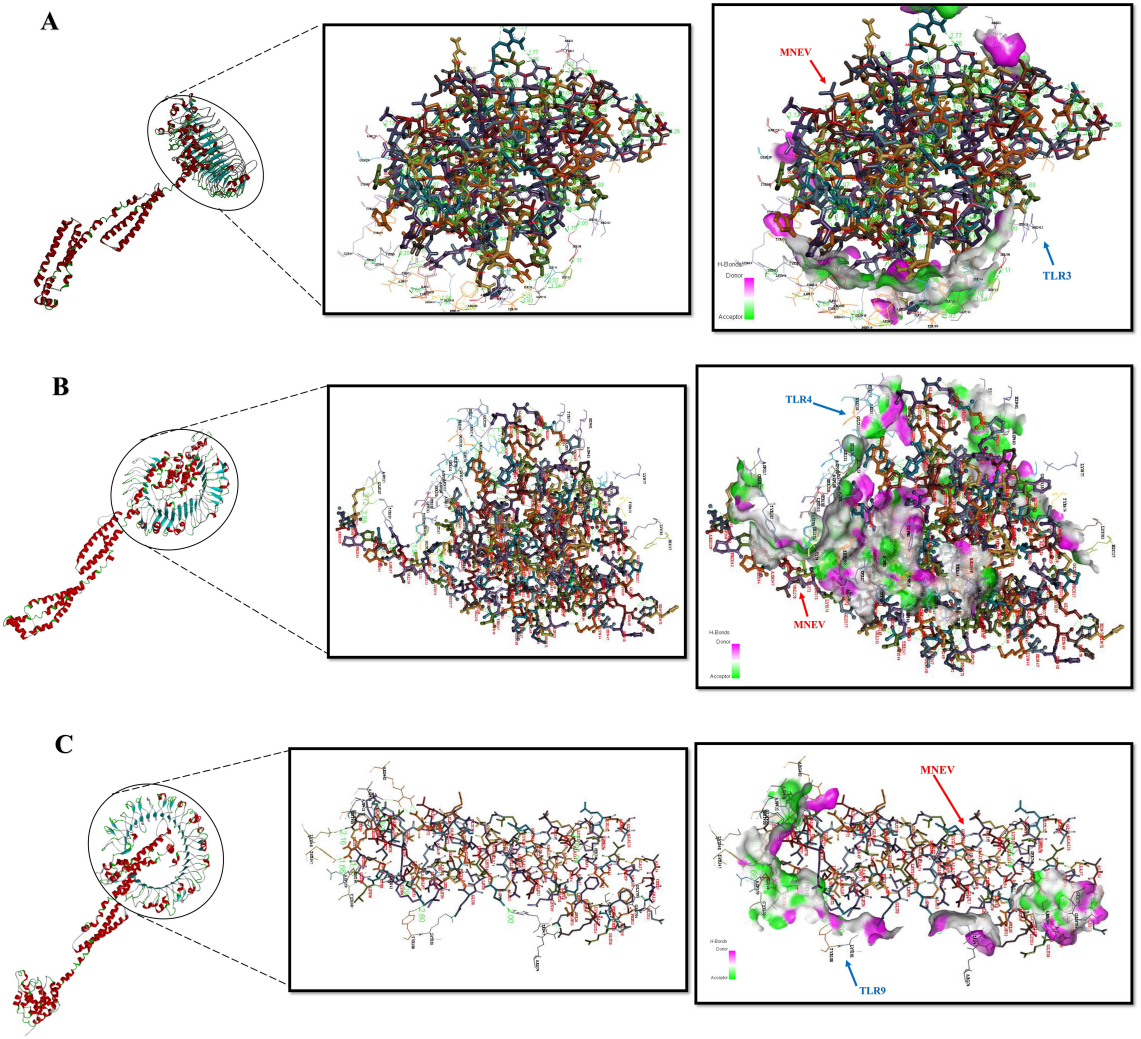
Protein-protein interactions analyzed using the ClusPro server. **(A)** Complex of the MNEV with TLR3. **(B)** Complex of the MNEV with TLR4. **(C)** Complex of the MNEV with TLR9. Hydrogen bonds are indicated.

Verifying the vaccine construct’s binding affinity with the immune receptor is essential to ensuring that it elicits the proper immune response. The route for the operation and control of the adaptive immune response is activated by the TLR proteins, which recognize pathogenic microbial components. The successful creation of peptide-based vaccines requires an understanding of the molecular specifics of antigen detection. The online ClusPro docking server was used to perform a docking simulation in order to examine the binding interactions between the developed MNEV and TLR3, TLR4, and TLR9. Cluster 0 was chosen for additional analysis because it had the lowest energy score (-924.8 (TLR3), -1043.8 (TLR4), and -958.5 (TLR9)) (**Table 2**, **Figure 6**). The MNEV-TLR3 complex created 41 hydrogen bonds, the MNEV-TLR4 complex formed 88, and the MNEV-TLR9 complex formed 24, according to the Discovery Studio visualizer (See **Supplementary Data S4**).

**Table 2:** Results of molecular docking analysis of the proposed MNEV sequence with Toll-like receptors TLR3, TLR4, and TLR9 using the ClusPro server.

TLR/MNEV	Server	Representative	Weighted Score
TLR-3 and MNEV	Cluspro	Center	-854.3
		Lowest Energy	-924.8
TLR-4 and MNEV	Cluspro	Center	-941.6
		Lowest Energy	-1043.8
TLR-9 and MNEV	Cluspro	Center	-958.5
		Lowest Energy	-958.5

### MM-GBSA binding free energy

The TLR and vaccine complex binding free energies were determined using the Molecular Mechanics Generalized Born Surface Area (MM-GBSA) approach. For TLR3, TLR4, and TLR9, the computed total MM-GBSA binding free energies were -86.4 kcal/mol, -313.26 kcal/mol, and -89.38 kcal/mol, respectively (**Table 3**). These results show that the MNEV and the corresponding TLRs have robust binding interactions; of the three receptors tested, TLR4 had the highest advantageous binding energy.

**Table 3:** Computed binding free energies of MNEV with TLR3, TLR4, and TLR9 using MM/GBSA methodology.

MM/GBSA	TLR3- MNEV	TLR4- MNEV	TLR9- MNEV
Binding free energy of complex	-86.4 (kcal/mol)	-313.26 (kcal/mol)	-89.38 (kcal/mol)

### *In silico* cloning and optimization of designed MNEV

The JCat optimized codons for high protein expression in eukaryotic organisms. For a MNEV with 472 aa, the ideal codon sequence length was 1416 nucleotides. The optimized nucleotide sequence’s CAI value was 0.99 and its CG-content was 53.4%, indicating a high likelihood of the recombinant vaccine being expressed in the host Eukaryotic Organisms. By supporting the BamHI and XbaI restriction enzymes, SnapGene software was utilized to introduce modified codon sequences into the Plenti-Giii-Cmv-Gfp-2A-Puro vector. 10844 bp make up the final product (vector and optimized codon sequence) (**Supplementary Data S4**).

### *In silico* immune simulation

**Figure 7:**
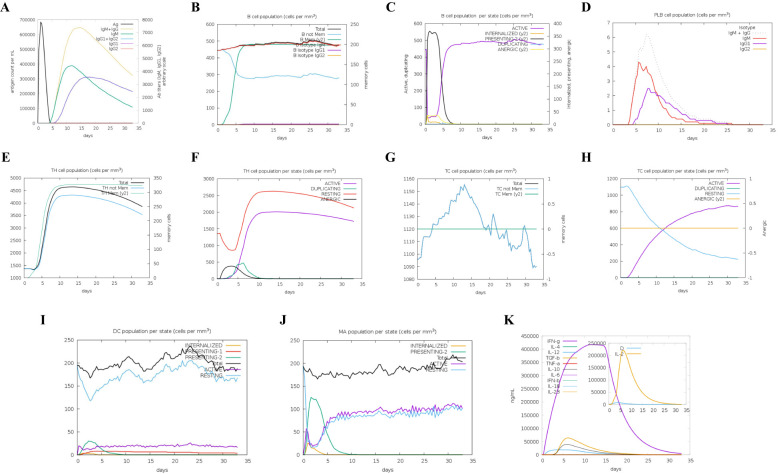
Immune simulation results. **(A)** Overview of both immunoglobulins and antigens, with antibodies categorized by their isotype. **(B)** B cell populations, including total counts, memory cells, and subdivisions into IgM, IgG1, and IgG2 isotypes. **(C)** Distribution of B lymphocytes by entity-state, displaying counts for active, class-II, internalized antigens, replicating, and anergic states. **(D)** Number of plasma B cells categorized by isotype (IgM, IgG1, and IgG2). **(E)** Total count of CD4 T-helper cells, including memory and overall counts. **(F)** CD4 T-helper cell counts broken down by entity-state (replicating, anergic, resting, and active). **(G)** Total number of CD8 T-cytotoxic cells, with memory and overall counts displayed. **(H)** CD8 T-cytotoxic cells categorized by entity-state. **(I)** Dendritic cells displaying antigenic peptides on MHC I and MHC II molecules; curves categorize the overall count into internalized, resting, active, and presenting states. **(J)** Total number of macrophages categorized as internalized, resting, and active on MHC II. **(K)** Concentration levels of interleukins and cytokines; the danger signal is indicated by “D” in the inset graphic.

To elucidate the development of adaptive immunity and immunological interactions, an *in silico* immune simulation study was conducted on the designed MNEV. This immunological simulation revealed a significant enhancement of the primary immune response with each incremental dosage, as evidenced by the progressive fluctuations in the levels of various immunoglobulins. This approach enabled a detailed examination of how immune responses evolve over time, providing insights into the dynamics of immunoglobulin production in response to vaccination. Additionally, there was an increase in the secondary immunological response (**Figure 7A**). Helper T-cells (**Figures 7E, F**), plasma B-cells (**Figure 7D**), active B-cells (**Figures 7B, C**), and regulatory and cytotoxic T cells (**Figures 7G, H**) were all seen to be growing in number. These findings suggest that a robust secondary immunological response, increased antigen clearance, and robust immune memory formation take place following each injection. Antigen presentation was enhanced, as shown by increased dendritic cell and macrophage density (**Figures 7I, J**). Additionally, the vaccine protein might generate a wide variety of cytokines (**Figure 7K**).

### Serum levels of total IgG increased by the MNEV

**Figure 8:**
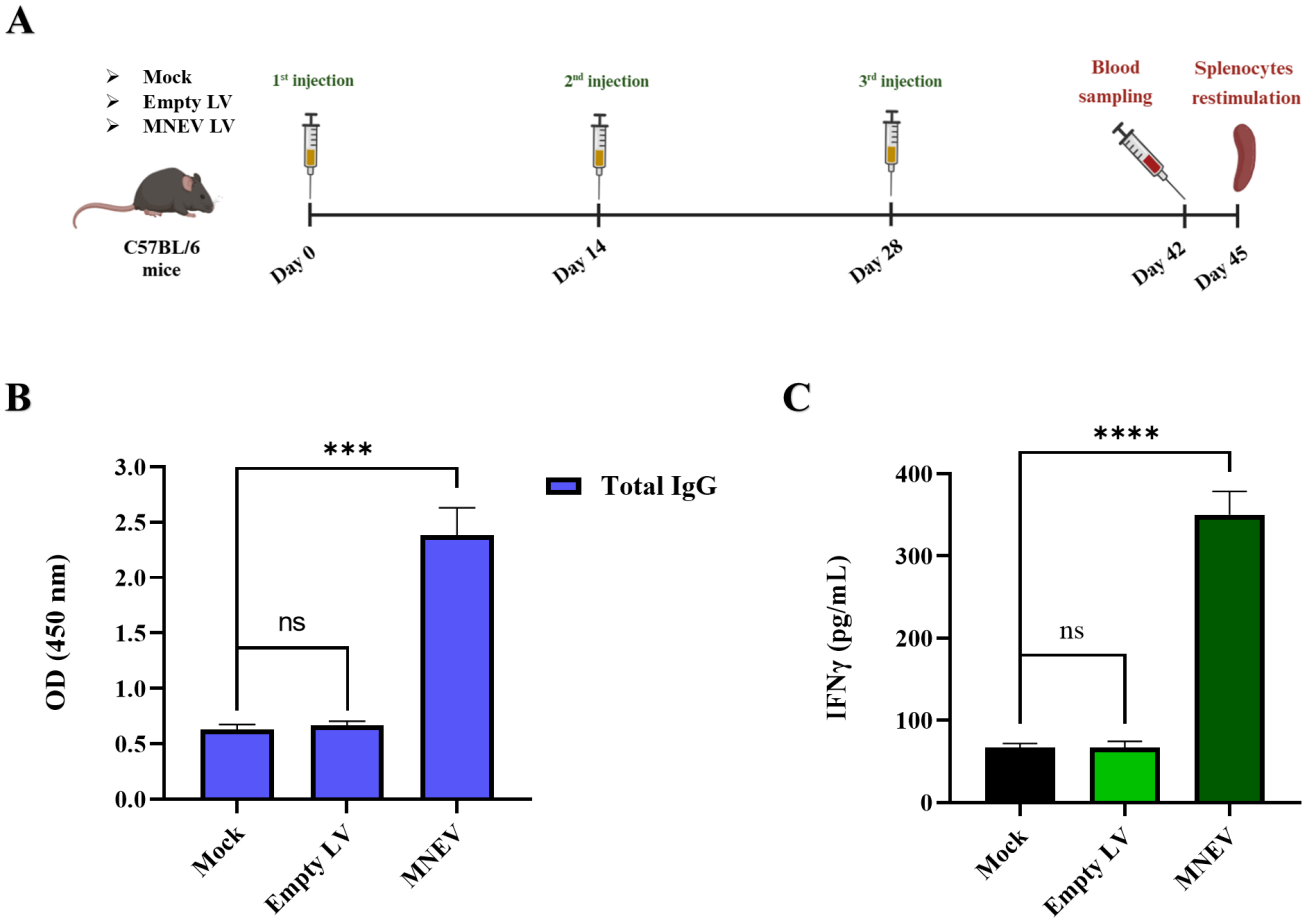
Anti-tumor effects of immunization with the designed MNEV in different study groups. **(A)** Overview of mouse immunization strategy and analysis schedule. **(B)** Serum levels of total IgG were determined by ELISA two weeks after the third injection across different groups. Notably, MNEV elicited a significant IgG response following the third injection. **(C)** The levels of IFN-γ secretion from splenocyte cultures after restimulation with MNEV were assessed using ELISA. Data are presented as means ± standard deviation (SD) for three mice per group. Statistical significance is denoted as follows: ns, not significant; ***P < 0.001; ****P < 0.0001. Mouse immunization workflow, illustrated by BioRender.

Two weeks after the final immunization, the total IgG levels in the serum of study groups were evaluated using ELISA. The results showed that serum levels of total IgG were significantly elevated in the MNEV immunization groups compared to those receiving the empty LV and Mock. No statistically significant difference was observed between the empty LV and Mock groups, indicating that the immunogenic effect was primarily attributed to the MNEV (**Figures 8A, B**).

### MNEV induced IFN-γ secretion

Spleens from the vaccinated animals were collected two weeks following the final injection, and splenocytes were subsequently cultured. The splenocytes derived from the MNEV-immunized groups demonstrated a significantly elevated secretion of IFN-γ cytokine compared to those from the Mock and empty LV control groups upon *in vitro* re-stimulation with MSC-MNEV-CM (**Figures 8A, C**). However, no significant differences in IFN-γ levels were observed between the splenocytes of the Mock and empty LV control groups.

### MNEV induces the CD19+ B cells and CD3+ T cells

**Figure 9:**
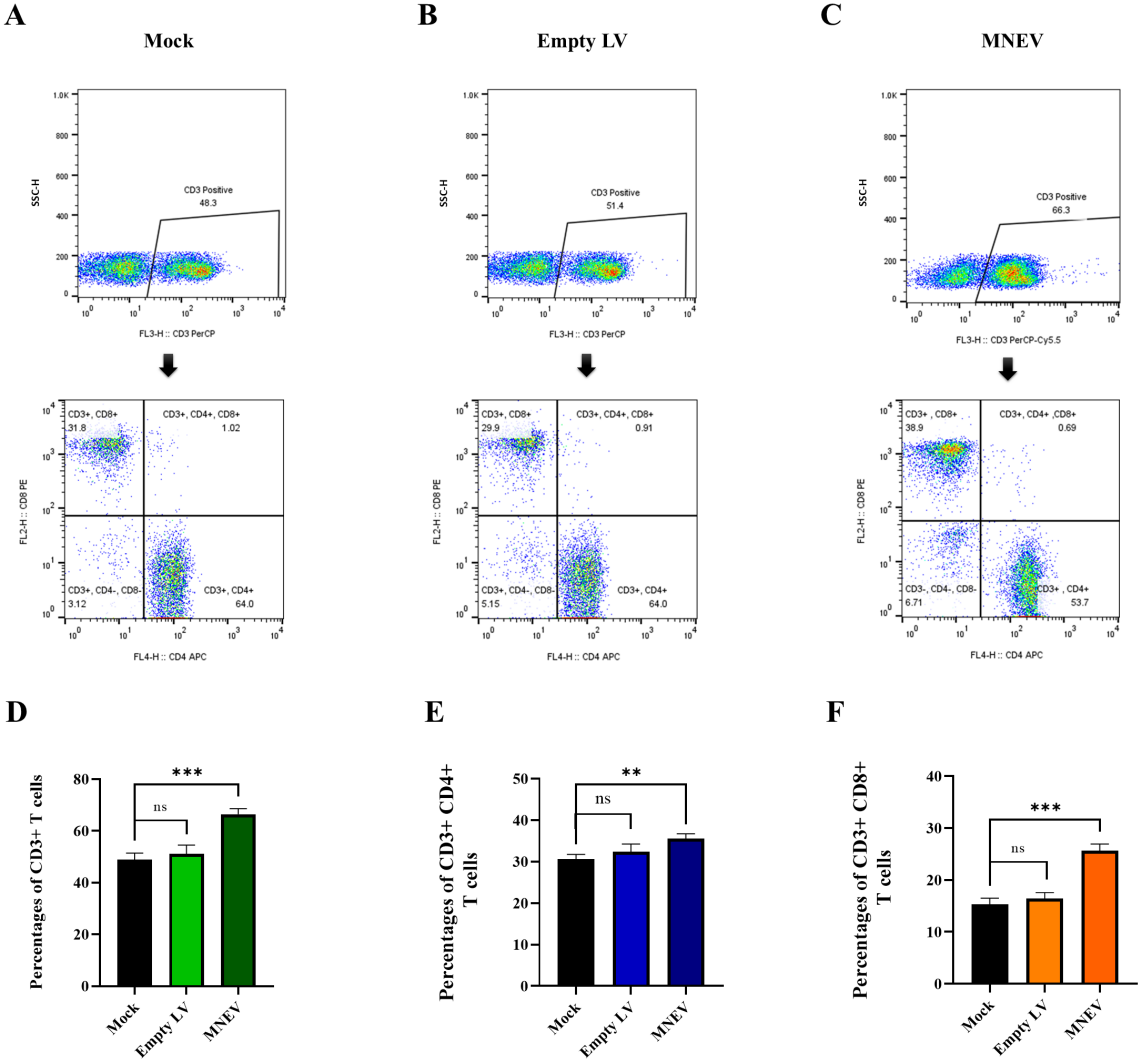
Flow cytometry analysis of CD3+ CD4+ and CD3+ CD8+ T cell levels in immunized mice. **(A–C)** Restimulation assays were performed using MNEV in splenocytes from study groups. CD3-positive cells were gated and analyzed for CD4 and CD8 markers. **(D–F)** Flow cytometry graphs represent data from restimulation assays in splenocytes of study groups. Data are presented as means ± standard deviation (SD). Statistical comparisons between the four groups were conducted using one-way ANOVA (ns, not significant; **p < 0.01, ***p < 0.001).

Flow cytometry was employed to examine alterations in CD3+ CD4+ and CD3+ CD8+ T lymphocyte populations. The data presented in **Figure 9** reveal that MNEV immunization led to a pronounced enhancement of both CD3+ CD4+ and CD3+ CD8+ T cell subsets among splenocytes, distinguishing it from other groups. Conversely, the Mock and empty LV groups showed no significant differences in the proportions of CD3+ CD4+ and CD3+ CD8+ T cells.

**Figure 10:**
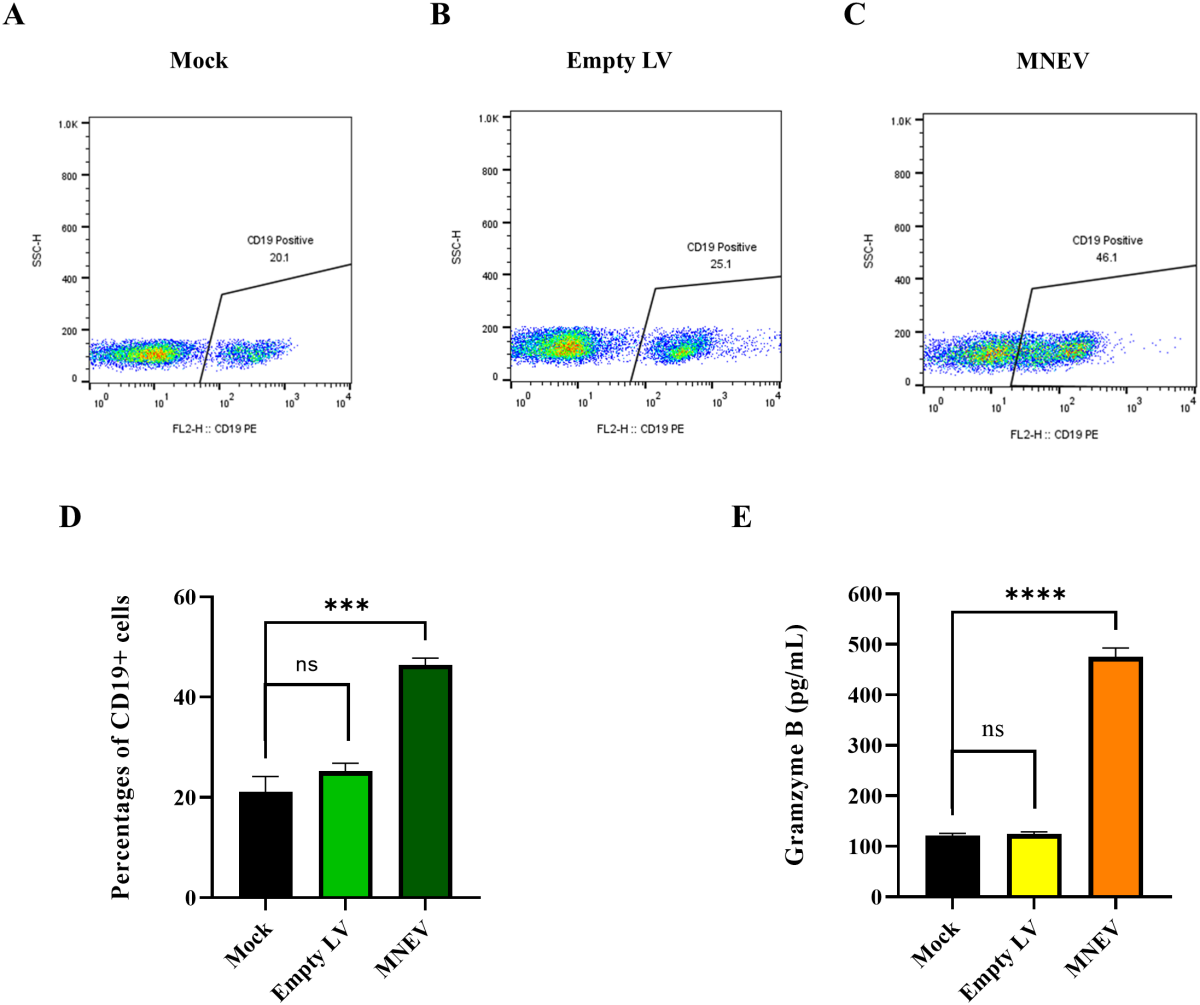
MNEV induction of CD19+ B cells and Granzyme B levels *in vitro*. **(A–C)** Flow cytometry plots of CD19+ B cells in splenocytes post-restimulation. **(D)** Histogram showing the percentages of CD19+ B cells following restimulation assays in splenocytes from study groups. **(E)** The levels of granzyme B secreted by splenocytes isolated from MNEV-immunized mice two weeks following the final immunization. The supernatants were collected from splenocytes that had been restimulated with MNEV. Granzyme B levels in splenocyte cultures were quantified using the ELISA technique. The data, representative of three independent experiments, are presented as mean values with standard deviations (SD). Notably, MNEV elicited significantly elevated Granzyme B levels compared to control groups, with statistical significance indicated by ns, not significant; ***p < 0.001 and ****P < 0.0001.

To investigate the impact of MNEV on the proportion of total B cells *in vitro*, splenocytes from MNEV-immunized mice were restimulated with MSC-MNEV-conditioned medium (CM) for 48 hours. The results indicate that, compared to the control group, MNEV significantly enhanced the proportion of CD19+ total B cells. In contrast, no significant differences between the Mock and empty LV control groups were observed (**Figures 10A–D**). These findings suggest that MNEV could effectively promote humoral and adaptive immune responses by stimulating the activation and proliferation of T and B cells.

### MNEV induced granzyme B secretion

To evaluate antigen-specific cytotoxic CD8+ T cell responses following vaccination, granzyme B levels in immunized mice were measured two weeks post-final immunization. The results presented in **Figure 10E** show that MNEV administration resulted in substantially higher granzyme B levels compared to control groups. In contrast, there were no statistically significant differences between the Mock and empty LV groups regarding granzyme B production. This indicates that MNEV could effectively induce robust cytotoxic T cell activity.

## Discussion

NSCLC accounts for approximately 85% of lung cancer cases and is a significant health concern due to its role as the primary cause of cancer-related deaths globally (83–85). Effective therapies are crucial given the five-year survival rate for NSCLC, which is often reported as around 15% to 28% depending on the source and stage of the disease, with survival rates decreasing significantly in later stages. Immunotherapy, which leverages the immune system to target cancer cells, offers a promising approach that may enhance lifespan and quality of life, particularly for patients with specific genetic abnormalities (83, 86). Subunit cancer vaccines that induce protective immune responses against tumor peptide antigens have been developed for cancer prevention or treatment. The feasibility of peptide-based vaccines has increased due to advancements in antigen identification methods, with various clinical trials (NCT05238558, NCT04701021, NCT04574583, NCT02455557, NCT00911560) showing encouraging outcomes (87).

Recent years have seen extensive research into cancer, including preventive strategies, novel vaccine development, and efforts to discover potential medications. *In silico* technologies have made substantial contributions due to their ability to provide rapid findings, which are essential for addressing such health crises. MNEVs are advantageous due to their safety, stability, ease of production, and ability to elicit both humoral and cellular immune responses (88).

By incorporating multiple epitopes from different tumor-associated antigens, MNEVs activate distinct T-cell subsets (CD4+ and CD8+) and B cells, producing a more potent and comprehensive response against cancer cells. The inclusion of epitopes restricted by MHC molecules enhances T-cell recognition and allows for a greater variety of T-cell responses, improving efficacy against diverse tumor antigen profiles. Adjuvants enhance immunogenicity and ensure a regulated immune response, fostering resilience without the risk of overactivation that could lead to autoimmunity (89, 90). By selecting epitopes less likely to cause adverse reactions, MNEVs reduce the likelihood of unfavorable immune responses compared to traditional vaccines, resulting in successful immunization with reduced toxicity (90, 91).

The neoepitope approach enables the creation of personalized treatments by identifying distinct mutations in each patient’s tumor, allowing for precise targeting of cancer cells while protecting healthy tissues, thereby enhancing treatment outcomes and reducing side effects (91, 92). These vaccines also promote long-lasting immunological memory, which is crucial for preventing cancer recurrence and ensuring the immune system remains vigilant against new tumor formations (90, 92). The potential of customized neoantigen treatment in NSCLC has been explored in several studies. Zhang et al. examined this prospect in 2021, focusing on effective immune responses from patient-specific mutations (93). A phase I study by Fenge Li in the same year found personalized neoantigen vaccination to be safe and viable for patients with advanced-stage NSCLC, particularly those with EGFR mutations (12). Heidary et al. developed a multi-neoantigen peptide vaccine using bioinformatics in 2022, highlighting its immunogenic properties for NSCLC treatment (89). Viborg and colleagues investigated DNA-based neoepitope vaccine in 2023, demonstrating its ability to elicit anti-tumor immunity in mouse models, supporting its use in customized immunotherapy (94). Additionally, 2024 research showed that a customized neoantigen vaccine enhances therapeutic effectiveness in advanced NSCLC when combined with current therapies like bevacizumab and anti-PD-1 antibodies (95).

In our study, we developed a customized MNEV targeting NSCLC using LL/2 lung cancer cells in a C57BL/6 mice model. The process began with whole exome sequencing (WES) and RNA sequencing (RNA-seq) to identify specific genetic variants linked to cancer that were consistent across six lung cancer datasets from the SRA. We prioritized gene mutations that were common and reproducible in all datasets. Based on this analysis, we constructed a personalized vaccine targeting 15 neoepitopes that elicit both cellular and humoral immune responses, making them ideal candidates for peptide vaccine design. By focusing on specific genetic targets in the LLC1 cell line and incorporating MNEV, our strategy aimed to enhance the vaccine’s specificity and effectively target the unique genetic alterations of the tumor. This approach not only potentially increases the vaccine’s efficacy by tailoring it to an individual’s genetic profile but also minimizes off-target effects, optimizing treatment outcomes.

To ensure the accuracy of neoepitope selection, we utilized *in silico* techniques with a focus on forecasting MHC I, MHC II, and linear and conformational B-cell neoepitopes. For MHCII predictions, we used MHC2pred, IEDB, RANKPEP, NetMHCIIpan-4.0 (BA), and NetMHCII 1.1 (SMM-Align). For MHCI predictions, we employed tools such as NetMHCPan 4.1 (BA), SYFPEITHI, NetCTLpan, and NetMHCcons. The ElliPro tool was used for structural neoepitopes in conjunction with ABCPred, BepiPred-2.0, and SVMTriP to identify linear neoepitopes that bind to BCRs. Neoepitopes were only chosen as final candidates if they satisfied the requirements of all pertinent instruments. 15 unique neoepitopes, including 5 MHC I-restricted, 4 MHC II-restricted, 4 linear B-cell, and 2 structural B-cell epitopes were found from the initial pool of 108 examined neoepitopes. It was anticipated that the chosen neoepitopes would have high binding affinities and be both immunogenic and antigenic, while also being non-toxic and non-allergenic. This comprehensive strategy highlights the importance of combining several prediction methods to improve the accuracy of neoepitope selection for vaccine development.

The inclusion of the 50S L7/L12 ribosomal protein as an adjuvant in lung cancer vaccine constructs was strategically significant for enhancing immune responses. This protein plays a crucial role in dendritic cell (DC) maturation by activating these cells through TLR4, leading to increased production of pro-inflammatory cytokines such as TNF-alpha, IL-1beta, and IL-6 (96). This maturation is essential for the effective activation of naïve T cells, thereby promoting a robust cellular immune response. Furthermore, studies indicate that combining L7/L12 with other immunogenic proteins can amplify immune responses, enhancing the overall efficacy of the vaccine (97, 98). The incorporation of L7/L12 should also be accompanied by assessments of cytokine profiles, as increased secretion of IFN-gamma from CD4+ and CD8+ T cells indicates a favorable Th1-type immune response, which is beneficial for anti-tumor immunity (96).

To connect distinct epitopes and maximize their presentation to the immune system, L7/L12 can be combined with a variety of linkers (including EAAAK, KK, and GPGPG). These linkers improve antigen presentation as an adjuvant, resulting in more robust and sustained immune responses. This is particularly significant for vaccines that target complicated diseases or malignancies. Crucially, L7/L12 is often regarded as non-toxic and non-allergenic, which lowers the possibility of negative responses in those who have received the vaccine. Its efficacy in MNEV for TB (99), and COVID-19 (100) has been shown in studies, underscoring its adaptability and function in enhancing immune responses (101). In our study, we used L7/L12 as an adjuvant to improve immune system activation.

When designing MNEVs, signal peptides play a crucial role in ensuring efficient vaccine component presentation and secretion, which results in a strong immune response (102–105). The Igκ signal peptide, in particular, guides proteins to the endoplasmic reticulum for appropriate folding and secretion (106, 107). It enhances recombinant protein secretion by efficiently directing proteins into the secretory pathway, ensuring proper translocation to the ER, correct folding, and post-translational modifications. This reduces intracellular accumulation, minimizes aggregation or degradation, and increases extracellular yield. Its proven effectiveness in various systems makes it a reliable choice for improving recombinant protein production (108, 109).

The selection of suitable linkers is critical in vaccine design, as defective linker selection can alter protein properties (110, 111). We used linkers like EAAAK, KK, GPGPG, and ADARY to join adjuvants, linear B-cell epitopes, and HTL and CTL neoepitopes in our NSCLC MNEV. These linkers enhance the overall immunogenicity of the vaccine and the integration of immunological components. For instance, KK facilitates B-cell identification and antibody formation (105), while EAAAK ensures appropriate distance between adjuvants and neoepitopes to promote successful immune responses (89, 112). GPGPG improves interactions with MHC II molecules, promoting T-cell activation (105), whereas ADARY preserves the structural integrity of CTL epitopes for efficient presentation to MHC I molecules (89, 112). Our strategy aligns with related research emphasizing the significance of specific linkers in maximizing immune responses against NSCLC while reducing off-target effects to enhance treatment outcomes.

Epitope selection was validated by docking individual neoepitopes with MHC I, MHC II, and BCRs. The final structure of the MNEV was aligned with TLR 3, 4, and 9 after confirming hydrogen bonding contacts. Interestingly, the link with the lowest energy showed a robust contact, indicating a possibility for successful binding. Khairkhah et al. presented the creation of a multi-epitope vaccine against COVID-19 that targets MHC I and MHC II in addition to structural proteins S, N, and M using BCR selection techniques. By encouraging efficient immune recognition and response, the final vaccine structure was tailored to interact with TLRs 2, 3, and 4, showing a significant connection that improves safety and efficacy (113). This strategy aligns with related research by Kumar et al., which examined the structural relationships between a multi-epitope vaccine and TLR-3, 7, and 8 (114). Another study developed a multi-epitope vaccine targeting important cancer antigens like STAT3, HER2, and GRB7 using a multimodal strategy that included MHC I, MHC II, CTL, and B-cell epitopes created from MAGE-A3, EGF, and MUC-1 by *in silico* immunoinformatics techniques. This vaccine integrated interactions with TLR2, TLR4, MHCI, and MHCII, showing high affinity for human receptors (115). Additionally, a similar study developed a multi-epitope peptide based on neoantigens that targets TLR4/MD2 as a possible vaccine against colorectal cancer, demonstrating its reliance on TLR4 binding and capacity to trigger powerful immune responses against colorectal cancer antigens (116). In a different study, the Silica Immunoinformatics approach was used to construct a multifunctional vaccine for NSCC cancer that included MHC I, MHC II, CTL, and B-cell epitopes obtained from MAGE-A3 cells, EGF, and MUC-1. Strong affinity between the vaccine and human receptors (TLR-2, TLR-4, MHCI, and MHCII) was found by molecular binding study (117). This comprehensive strategy emphasizes the importance of combining different immunoinformatics techniques when creating vaccines that effectively elicit strong immune responses against cancer and infectious illnesses. Our study’s conclusions offer important new information on current MNEV development efforts. By using immunoinformatics to create a safe and tailored immunotherapy, this work demonstrates the potential of customized MNEV for NSCLC. By carefully choosing neoepitopes and creatively using adjuvants and linkers, we created a construct that is highly immunogenic and selective while reducing off-target effects. Strong interactions with immune receptors were validated by molecular modeling, highlighting the vaccine’s potential for long-lasting immune response and efficient tumor targeting. These discoveries advance the science of cancer immunotherapy by providing a route for specialized, accurate therapies against lung cancer and other cancers.

Our preclinical data in a mouse model demonstrate that the MNEV induces a significant immune response. The significant elevation of serum IgG levels, increased proportions of CD19+ B cells, and enhanced CD3+ CD4+ and CD3+ CD8+ T-cell populations in MNEV-immunized mice indicate a strong activation of both humoral and adaptive immunity. Furthermore, the marked increase in IFN-γ and granzyme B secretion by splenocytes from MNEV-immunized mice underscores the vaccine’s capacity to stimulate cytotoxic T-cell activity, a critical mechanism for tumor cell elimination. The absence of similar responses in control groups confirms the MNEV’s specific effect. The inclusion of the 50S L7/L12 ribosomal protein as an adjuvant and the Igκ chain signal peptide likely contributed to these effects by boosting immunogenicity and secretion efficiency, respectively, highlighting the value of strategic vaccine design.

However, the study has limitations, including the use of a mouse model and a small cohort size, which may not fully reflect human NSCLC’s complexity. Additionally, the lack of long-term data means that the durability of the immune response is unknown. To advance this approach, future research should employ larger animal models that better mimic human disease, conduct long-term studies to assess the vaccine’s lasting efficacy, and explore combination therapies to optimize treatment outcomes. For translating this approach to human NSCLC, the development of personalized MNEVs based on patient-specific neoantigens is essential. To test these human-specific MNEVs, future research could utilize humanized mouse models, which have human immune systems, to assess their efficacy in a preclinical setting. Additionally, long-term studies are necessary to evaluate the durability of the immune response and the vaccine’s ability to prevent or delay tumor recurrence. Exploring combination strategies with existing therapies, such as chemotherapies and immunotherapies, and optimizing vaccine delivery methods will be crucial to bring this promising approach to clinical practice.

## Conclusions

Our study presents the development and evaluation of a MNEV designed using reverse vaccinology and bioinformatics approaches for targeting NSCLC. By focusing on neoepitopes derived from tumor-specific mutations, our vaccine aims to elicit a precise immune response against cancer cells while minimizing off-target effects. Leveraging whole exome sequencing and RNA sequencing data, we identified and validated neoepitopes that stimulate B cells, HTLs, and CTLs. The incorporation of the 50S L7/L12 ribosomal protein as an adjuvant and the Igκ chain signal peptide enhanced immune responses and secretion efficiency, respectively. Our *in-silico* evaluations confirmed the vaccine’s non-toxicity, non-allergenicity, and stability, with high-affinity interactions with immune receptors. Immunization with the MNEV in a mouse model resulted in significant increases in serum IgG levels, CD19+ B cells, and CD4+ and CD8+ T cells, along with enhanced IFN-γ and granzyme B secretion. These findings highlight the MNEV’s potential as a promising immunotherapeutic strategy for NSCLC. Our study contributes to the growing field of neoepitope-based vaccines and provides a robust framework for the design and evaluation of such vaccines in cancer immunotherapy, building on and extending previous research in multi-epitope vaccine development.

## Data Availability

The datasets presented in this study can be found in online repositories. The names of the repository/repositories and accession number(s) can be found in the article/**Supplementary Material**.
