# Supplementary material for: Development of a multi-neoepitope vaccine targeting non-small cell lung cancer through reverse vaccinology and bioinformatics approaches
Source: Front Immunol. 2025 May 16;16:1521700. doi: 10.3389/fimmu.2025.1521700 (PMC12122770; doi:10.3389/fimmu.2025.1521700)
Supplement: Supplementary file 6 [file DataSheet6.docx]

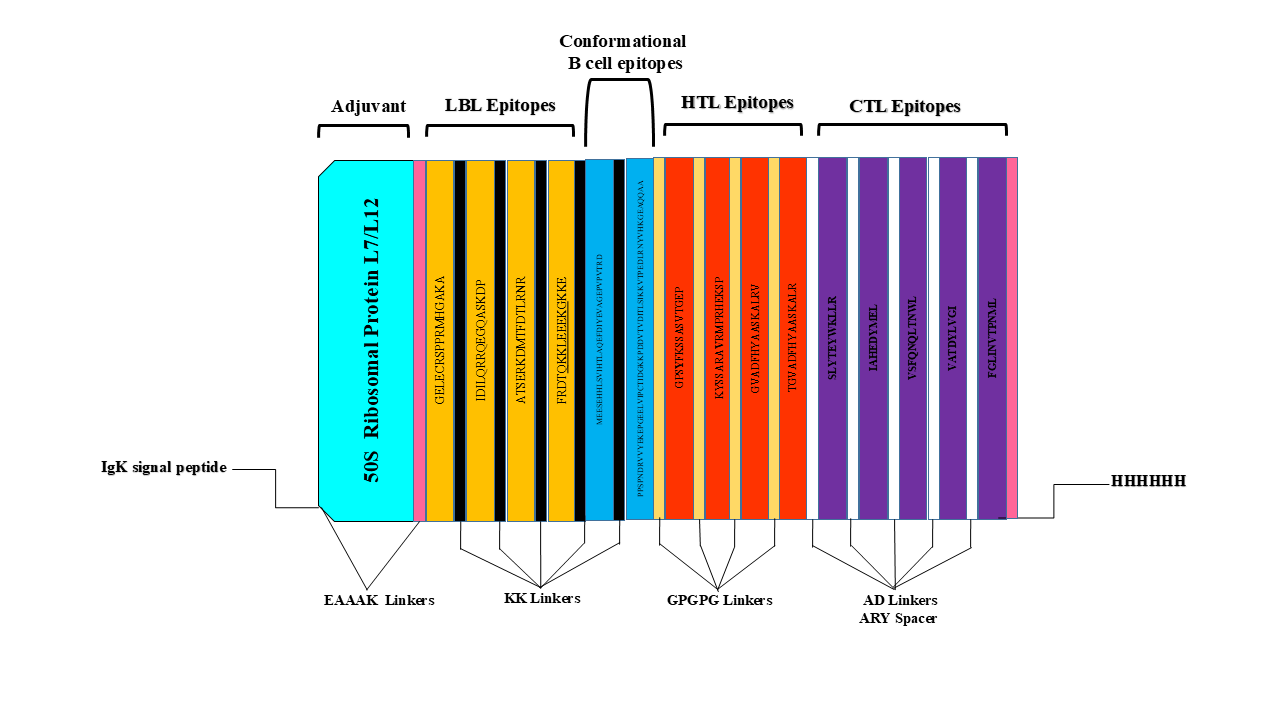


**Fig F1**. **Visual representation of MNEV design.** This figure illustrates the design of a MNEV strategy. From left to right, the MNEV components include LBL, conformational B cell epitopes, HTL (Helper T cell) epitopes, and CTL (Cytotoxic T cell) epitopes, all accompanied by an adjuvant. Each component is displayed in rectangular boxes labeled as follows: "Green Abbey" for the adjuvant, "Orange" for LBL epitopes, "Blue" for conformational B cell epitopes, "Red" for HTL epitopes, and "Purple" for CTL epitopes. The EAAAK linker (Pink) connects the adjuvant to the first LBL epitope. KK linkers (Black) are utilized to join the LBL epitopes together. GPGPG linkers (Orange) facilitate the connection between HTL epitopes, while AD/ARY linkers (White) are employed to link the CTL epitopes.


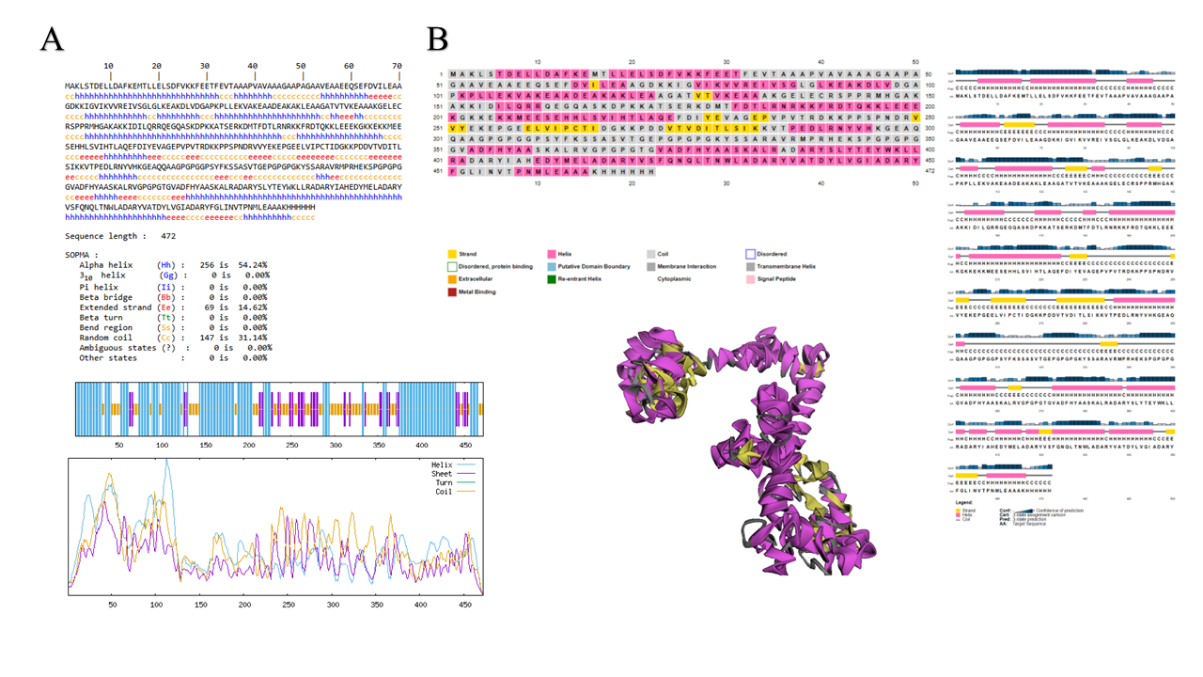


**Fig F2**. **Visualization of the Secondary Structure of the Designed MNEV and Identification of Alpha Helix and Beta Sheet Regions Using Prabi and PSIPRED Servers.** This figure presents the secondary structure analysis of the designed vaccine, highlighting the identification of alpha helices and beta sheets. **(A)** The output from the Prabi server displays the predicted secondary structural elements, with alpha helices represented in [specific color] and beta sheets in [specific color]. **(B)** The results from the PSIPRED server further validate these predictions, offering a detailed representation of the secondary structural features. This visualization is crucial for understanding the structural characteristics of the MNEV, as these elements are fundamental to its immunogenicity and functionality.


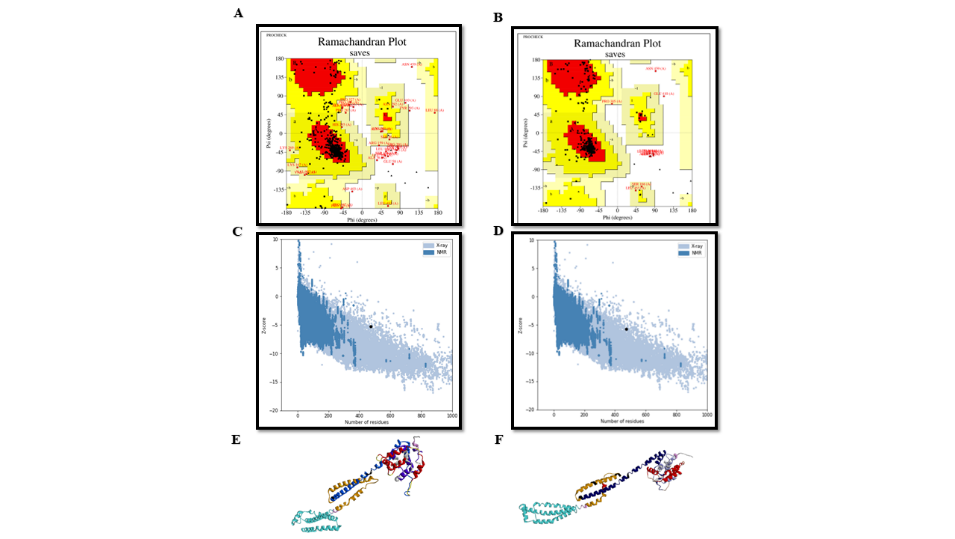


**Fig F3**. **Tertiary Structure Modeling, MNEV Construction Improvement, and Validation.** This figure presents the modeling and validation of the tertiary structure of the designed MNEV. (A) Ramachandran plot before refinement and (B) after refinement. These plots illustrate the distribution of dihedral angles (phi and psi) of amino acid residues, indicating the conformational quality of the modeled structure. The refinement process is expected to enhance the proportion of residues in favored regions while reducing those in disallowed regions. (C) Z-score graph generated by the ProSA online server for the modeled structure before refinement and (D) after refinement. This graph compares the modeled 3D structure to X-ray crystallographic structures of proteins of similar sizes, providing a quantitative assessment of model quality. (E, F) 3D structural representations of the vaccine model before and after refinement. These visualizations highlight improvements in structural integrity and stability achieved through the refinement process.


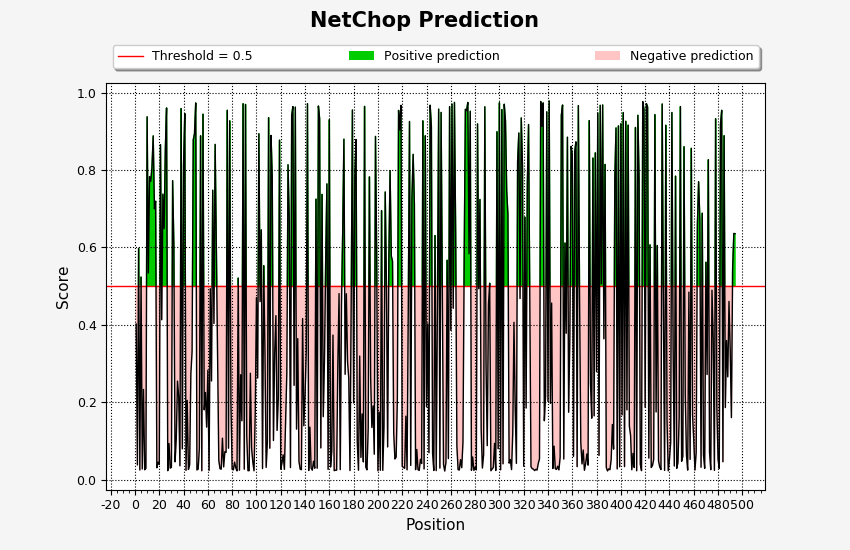


**Fig F4**. **Cleavage Sites.** This figure illustrates the identified cleavage sites and the corresponding prediction thresholds for the analyzed peptides. Positive predictions, indicating potential cleavage sites, are marked in green (n=172). In contrast, regions that fall below the threshold value of 0.5 are highlighted in pink, signifying areas with insufficient confidence for cleavage prediction. This visualization aids in distinguishing between reliable cleavage sites and those that require further investigation.


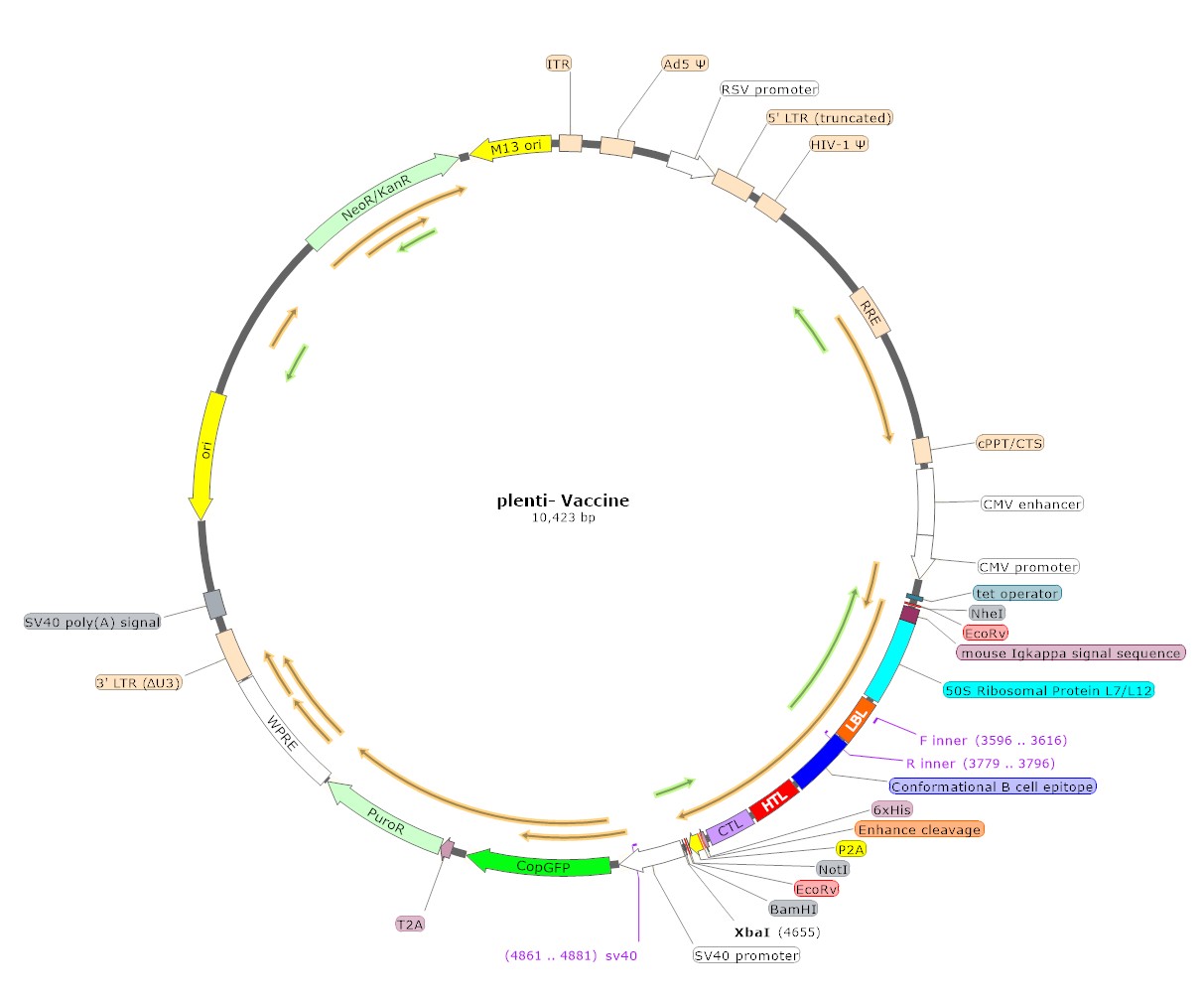


**Fig F5. *In Silico* Cloning of the MNEV Sequence Using SnapGene Software Free Trial.** The nucleotide sequence of the MNEV is highlighted, while the remaining sections of the circular diagram represent the backbone of the Plenti-Giii-Cmv-Gfp-2A-Puro vector.

**Table T1: Types of number of variants observed in all data.**

| **BioProject**  **Mutation types** | **PRJNA401728** | **PRJNA759882 (Day 7)** | **PRJNA786566 (6h)** | **PRJNA786566 (24h)** | **PRJNA759882 (Day 14)** | **PRJNA759882 (Day 21)** | **PRJNA1201561** |
| --- | --- | --- | --- | --- | --- | --- | --- |
| **Cell line** | **LLC1** | | | | | | **A549** |
| **Substitution (**missense) | 962 | 962 | 2201 | 2201 | 2201 | 2201 | 2003 |
| **Silent** | 630 | 633 | 1932 | 1932 | 1932 | 1932 | 2399 |
| **Noncoding Transcript** | 604 | 615 | 1436 | 1436 | 1436 | 1436 | 32760 |
| **Intronic** | 2652 | 2677 | 6753 | 6753 | 6753 | 6753 | 98849 |
| **Intergenic** | 383 | 387 | 907 | 907 | 907 | 907 | 67568 |
| **Five Prime UTR** | 88 | 89 | 251 | 251 | 251 | 251 | 1599 |
| **ThreePrimeUTR** | 220 | 220 | 534 | 534 | 534 | 534 | 4034 |
| **Premature Stop** | 44 | 45 | 81 | 81 | 81 | 81 | 25 |
| **Incomplete Transcript** | 15 | 15 | 54 | 54 | 54 | 54 | 1854 |
| **IntronicSpliceSite** | 40 | 40 | 100 | 100 | 100 | 100 | 106 |
| **Exonic Splice Site** | 3 | 3 | 7 | 7 | 7 | 7 | 7 |
| **SpliceDonor** | 6 | 6 | 15 | 15 | 15 | 15 | 10 |
| **Splice Acceptor** | 9 | 9 | 23 | 23 | 23 | 23 | 6 |
| **Deletion** | 2 | 2 | 4 | 4 | 4 | 4 | 0 |
| **Alternate Start Codon** | 1 | 1 | 3 | 3 | 3 | 3 | 1 |
| **Start Loss** | 1 | 1 | 1 | 1 | 1 | 1 | 8 |
| **Stop Loss** | 2 | 2 | 4 | 4 | 4 | 4 | 2 |
| **Frame Shift** | 20 | 20 | 44 | 44 | 44 | 44 | 0 |
| **Insertion** | 3 | 3 | 5 | 5 | 5 | 5 | 0 |
| **Frame Shift Truncation** | 1 | 1 | 1 | 1 | 1 | 1 | 0 |

## **Table T2. Interaction Similarity Scores of Selected CTL, HTL, and BCR Epitopes Using GalaxyPepDock, HPEPDOCK, and CABS Flexible Docking Server.**

| Epitope | Gene names | ALLELE | HPEPDOCK | | Galaxypepdock | | CABS | |
| --- | --- | --- | --- | --- | --- | --- | --- | --- |
|  | **Variant** |  | **Reference structure**  **(PDB ID: CHAIN)** | **Docking Score**  **(kcal/mol)** | **TM**  **Score**  **(> 0.6)** | **Estimated accuracy (>0.8)** | **Number of elements** | **Cluster density** |
| SLYTEYWKLLR | Rab3gap2  (chr1 g.185263697G>T) | H-2-Kb  (4PV8)  (4PV9) | 4PV9: A | -210.241 | 0.967 | 1.000 | 105 | 41.5889 |
| IAHEDYMEL | Thoc5  (chr11 g.4922008A>G) | H-2-Kb  (4PV8)  (4PV9) | 4PV9: A | -188.351 | 0.935 |  | 96 | 39.19 |
| VSFQNQLTNWL | D930048N14Rik  (chr11 g.51651161C>A) | H-2-Db  (7N9J) | 3FTG: A | -211.788 | 0.979 | 1.000 | 127 | 31.5801 |
| VATDYLVGI | Slc4a7  (chr14 g.14773295T>C) | H-2-Kb  (4PV8)  (4PV9) | 4PV9: A | -203.794 | 0.937 | 1.000 | 122 | 25.7772 |
| FGLINVTPNML | Dhrs9  (chr2 g.69394406T>C) | H-2-Db  (7N9J) | 3FTG: A | -217.229 | 0.979 | 1.000 | 127 | 44.8497 |
| GPSYFKSSASVTGEP | Nup214  (chr2 g.31994685C>A) | I-Ab  (4P23) | 4P23:C | -212.431 | 0.962 | 0.851 | 150 | 38.3118 |
| KYSSARAVRMPRHEKSP | Ric1  (chr19 g.29579868A>T) | I-Ab  (4P23) | 4P23:C | -212.706 | 0.952 | 0.819 | 114 | 42.3709 |
| GVADFHYAASKALRV | Epg5  (chr18 g.77986475A>G) | I-Ab  (4P23) | 4P23:C | -214.288 | 0.913 | 0.834 | 110 | 44.0731 |
| TGVADFHYAASKALR | Epg5  (chr18 g.77986475A>G) | I-Ab  (4P23) | 4P23:C | -221.857 | 0.913 | 0.834 | 100 | 135.836 |

**Table T3. List of amino acids involved in hydrogen bonds between peptide and MHC/BCR (galaxypepdock).**

| Ligand | Receptor | Hbond | | Distance |
| --- | --- | --- | --- | --- |
| SLYTEYWKLLR | H-2-Kb (**4PV8)** | GLU773:OE2 | LYS66:HZ1 | 1.88295 |
|  |  | **TYR771:O** | ASN70:HD21 | 1.96606 |
|  |  | THR772:OG1 | ARG755:HH21 | 2.42491 |
|  |  | TRP775:O | ASN760:HN | 2.13927 |
|  |  | TRP775:O | ASN760:HD22 | 2.13983 |
|  |  | GLU773:H | LYS66:O | 2.23457 |
|  |  | TYR774: HH | ASP77:OD2 | 1.95898 |
|  |  | TRP775:H | PHE758:O | 1.85776 |
|  |  | ARG779:HH11 | ILE761:O | 2.01663 |
|  |  | ARG779:HH11 | ILE761: OXT | 2.48212 |
|  |  | ARG779:HH12 | TYR84: OH | 2.47794 |
|  |  | ARG779:HH22 | ILE761:O | 2.36465 |
|  |  | ARG779:HH22 | ILE761: OXT | 2.27198 |
|  |  | GLU773:OE2 | LYS66:HZ1 | 1.88295 |
|  |  | THR772:OG1 | ARG755:HH21 | 2.42491 |
|  |  | TRP775:O | ASN760:HN | 2.13927 |
|  |  | TRP775:O | ASN760:HD22 | 2.13983 |
|  |  | SER769:H1 | TYR159:O | 2.07089 |
|  |  | SER769:H1 | CYS164:SG | 2.49885 |
|  |  | THR772:HG1 | GLU63:OE2 | 2.40951 |
|  |  | GLU773:H | LYS66:O | 2.23457 |
|  |  | TYR774: HH | ASP77:OD2 | 1.95898 |
|  |  | TRP775:H | PHE758:O | 1.85776 |
|  |  | ARG779:HH11 | ILE761:O | 2.01663 |
|  |  | ARG779:HH11 | ILE761: OXT | 2.48212 |
|  |  | ARG779:HH12 | TYR84: OH | 2.47794 |
|  |  | ARG779:HH22 | ILE761:O | 2.36465 |
|  |  | ARG779:HH22 | ILE761: OXT | 2.27198 |
| IAHEDYMEL | H-2-Kb (**4PV8)** | GLU772:OE2 | LYS66:HZ1 | 1.93409 |
|  |  | GLU772: N | ASN70:HD21 | 2.17705 |
|  |  | GLU772:O | ASN70:HD21 | 2.10943 |
|  |  | LEU777:O | LYS146:HZ3 | 2.22137 |
|  |  | GLU772:OE1 | TYR159: HH | 2.03318 |
|  |  | ALA770:O | PHE756:H | 2.29268 |
|  |  | MET775:O | ASN760:H | 1.99722 |
|  |  | LEU777:O | ASN760:HD22 | 2.03244 |
|  |  | ILE769:H2 | ARG755:O | 1.88383 |
|  |  | **ALA770:H** | **ARG755:O** | **2.03827** |
|  |  | HIS771:H | GLU24:OE2 | 1.93274 |
|  |  | HIS771:ND1 | GLU24:OE1 | 3.1933 |
|  |  | HIS771:ND1 | TYR45: OH | 2.75398 |
|  |  | GLU772:H | PHE756:O | 2.10835 |
|  |  | TYR774: HH | ASP77:OD2 | 1.90486 |
|  |  | MET775:H | PHE758:O | 1.99259 |
| VSFQNQLTNWL | H-2-Db(**7N9J)** | **THR402:OG1** | **ARG289:HH21** | **1.99888** |
|  |  | VAL395:H3 | GLU62:OE2 | 1.95591 |
|  |  | SER396: HG | GLY287:O | 2.43205 |
|  |  | GLN398:HE21 | GLY287:O | 1.93356 |
|  |  | ASN399:HD21 | GLU162:OE2 | 2.16348 |
|  |  | ASN399:HD21 | TYR277: OH | 2.22974 |
|  |  | ASN403:H | VAL282:O | 1.97881 |
| VATDYLVGI | H-2-Kb (**4PV8)** | **THR771:OG1** | ASN70:HD22 | 2.16845 |
|  |  | TYR773:O | GLN114:HE22 | 1.95106 |
|  |  | ASP772:OD1 | ARG755:H2 | 2.31881 |
|  |  | VAL769:O | ARG755:HH11 | 2.04488 |
|  |  | ALA770:O | PHE756:H | 2.22885 |
|  |  | ASP772:O | PHE756:H | 2.34173 |
|  |  | ASP772:H | ARG755:O | 1.89377 |
|  |  | GLY776 | ALA759:O | 2.0335 |
| FGLINVTPNML | H-2-Db(**7N9J)** | ILE398 | ARG280:HN | 2.41982 |
|  |  | PHE395:O | GLY287:HN | 2.44428 |
|  |  | GLY396:O | PHE288:HN | 2.01215 |
|  |  | **PHE395:H2** | GLU62:OE1 | 2.37368 |
|  |  | GLY396:H | TYR44: OH | 2.30453 |
|  |  | LEU397:H | TYR44: OH | 2.30631 |
|  |  | ILE398:H | GLY278:O | 1.84721 |
| GPSYFKSSASVTGEP | **I-Ab** | TYR384: HH | ASP56:O | 2.25917 |
|  |  | **TYR384: HH** | ASP56:O | 2.25917 |
|  |  | LYS386:HZ2 | ASP56:OD2 | 2.06787 |
|  |  | LYS386:HZ3 | GLN58:OE1 | 1.92714 |
|  |  | TYR384: HH | ASP56:O | 2.25917 |
|  |  | LYS386:HZ2 | ASP56:OD2 | 2.06787 |
|  |  | LYS386:HZ3 | GLN58:OE1 | 1.92714 |
| KYSSARAVRMPRHEKSP | **I-Ab** | LYS381:O | GLN183:HE21 | 2.07272 |
|  |  | **VAL388:O** | **LYS189:HN** | **2.02703** |
|  |  | PRO397: OXT | ARG243:HH12 | 1.98654 |
|  |  | PRO397: OXT | ARG243:HH22 | 2.16425 |
|  |  | PRO391:O | TYR248: HH | 2.48015 |
|  |  | LYS381:HZ1 | GLU181:OE2 | 2.01631 |
|  |  | LYS381:HZ3 | ASP56:OD2 | 2.04185 |
|  |  | TYR382: HH | ASP56:O | 2.04333 |
|  |  | SER383:HN | GLN183:OE1 | 1.99754 |
| GVADFHYAASKALRV | **I-Ab** | ALA383:O | GLN183:HE22 | 2.1619 |
|  |  | TYR387:O | LYS184:HZ2 | 2.04242 |
|  |  | ALA392:O | LYS189:HZ1 | 2.0533 |
|  |  | **GLY381:HT2** | HIS269:NE2 | 2.26804 |
|  |  | SER390: HG | ASN188:OD1 | 1.9247 |
|  |  | LEU393:HN | TYR248: OH | 2.10959 |
| TGVADFHYAASKALR | **I-Ab** | GLN183:HE21 | HIS387:NE2 | 2.42507 |
|  |  | LYS184:HZ1 | TYR388: OH | 2.42568 |
|  |  | LYS189:H | ALA390:O | 2.17125 |
|  |  | ASP192:H | LEU394:O | 2.14425 |
|  |  | HIS269:ND1 | THR381:OG1 | 2.84405 |
|  |  | HIS269:ND1 | ASP385:OD2 | 3.08467 |
|  |  | GLU272:OE1 | THR381:H1 | 1.95031 |
|  |  | **GLY59:O** | TYR388: HH | 1.9262 |
|  |  | GLU254:OE1 | LYS392:HZ1 | 1.95234 |
|  |  | ASP192:OD2 | ARG395: HE | 2.20953 |
|  |  | ASP192:OD2 | ARG395:HH21 | 1.92072 |
| GELECRSPPRMHGAKA | **BCR** | GLN265:HE21 | GLY298:O | 2.10207 |
|  |  | LYS283:HZ2 | GLU289: OE2 | 2.29825 |
|  |  | LEU288:H | ASP284:OD1 | 2.16322 |
|  |  | GLU289 | ASP284:OD1 | 1.9282 |
|  |  | CYS290:H | ASP284:OD2 | 2.33773 |
|  |  | CYS290:SG | ASP284:OD2 | 2.87016 |
|  |  | ARG295 | CYS122:O | 2.0398 |
|  |  | LYS300:H | GLN265:OE1 | 2.06556 |
| IDILQRRQEGQASKDP | **BCR** | ILE286 | ASP282:OD2 | 2.96308 |
|  |  | GLN290 | ALA123:O | 2.53081 |
|  |  | **GLY127:CA** | GLN290:OE1 | 3.55192 |
|  |  | GLN290:HE21 | ALA123:O | 2.53081 |
|  |  | GLY127:CA | GLN290:OE1 | 3.55192 |
|  |  | ILE286:H1 | ASP282:OD2 | 2.96308 |
|  |  |  |  |  |
| ATSERKDMTFDTLRNR | **BCR** | THR297:O | GLN265:HE21 | 2.0529 |
|  |  | SER288:O | LYS283:HZ2 | 2.1673 |
|  |  | SER288 | LYS283:HZ3 | 2.73178 |
|  |  | THR287:HG1 | ASP284:OD1 | 2.47937 |
|  |  | LEU298:H | GLN265:OE1 | 2.99766 |
|  |  | **ARG299: HE** | ILE111:O | 2.20332 |
|  |  | **ARG299:HH22** | ILE111:O | 2.56337 |
|  |  | ASN300:HD21 | LYS258:O | 2.56575 |
|  |  | ARG301:HH11 | ASN109:OD1 | 2.11146 |
|  |  | ASN300:O | LYS258:CA | 3.2257 |
|  |  | THR297:C | GLU115:O | 3.46481 |
|  |  | LEU298:CA | GLU115:OE2 | 2.98104 |
|  |  | LEU298:C | GLN265:OE1 | 2.99766 |
|  |  | ALA286:H2 | TRP136 | 2.98959 |
|  |  | ARG290:HH22 | VAL125:O | 2.34288 |
|  |  | THR297:O | GLN265:HE21 | 2.0529 |
| FRDTQKKLEEEKGKKE | **BCR** | **GLU301:OE2** | ARG253: HE | 2.31848 |
|  |  | **GLU301:OE1** | ARG253:HH21 | 2.26719 |
|  |  | **GLU301:OE2** | ARG253:HH21 | 1.9036 |
|  |  | **GLU301:OE1** | ARG254: HE | 1.94144 |

**Table T4. Physicochemical Parameters of MNEV Computed Using the ProtParam Tool.**

| Physicochemical properties | MNEV |
| --- | --- |
| Number of amino acids | 472 |
| Molecular weight | 51447.30 |
| Theoretical pI | 6.46 |
| Instability (less than 40) | 31.97 |
| Estimated half-life (*Escherichia coli*, *in vivo*) | 30 h (mammalian reticulocytes *in vitro*)   > 20 h (yeast, *in vivo*)   > 10 h (Escherichia coli *in vivo*) |
| Aliphatic index (range of 66.5 to 84.33) | 74.53 |
| Grand average of hydropathicity (GRAVY) = -2 to +2 | -0.546 |
| solubility of the vaccine construct (equal to or more than 0.5) | 0.749403 |
| Protein-Sol online server (>0.45) | 0.656 |
| Net charge at pH 7 | *-2.6* |
| Estimated solubility: | Good water solubility*.* |

**Table** **T5. Identification of Predicted Neoantigens Their Proteasomal in NetChop Data.** This table presents the identification of predicted neoantigens associated with the identified cleavage sites, along with their corresponding proteasomal scores derived from the NetChop analysis. The data indicate that these neoantigens exhibit excellent proteasome, highlighting their high potential for effective antigen processing and presentation.

| Neoepitope-Mouse MHCI | Proteasome score  (Threshold   =0.5 / Prediction method=c term 3.0) |
| --- | --- |
| SLYTEYWKLLR | **0.726406** |
| IAHEDYMEL | **0.891515** |
| VSFQNQLTNWL | **0.919575** |
| VATDYLVGI | **0.934709** |
| FGLINVTPNML | **0.965003** |

**Table T6. List of Amino Acids Involved in Hydrogen Bonds Between the MNEV and TLR3, TLR4, and TLR9**. This table presents the hydrogen bond interactions between TLRs and MNEV molecules, along with the distances measured in Angstroms (Å).

| Ligand | Receptor | Hbond | | Distance |
| --- | --- | --- | --- | --- |
| MNEV | **TLR-4** |  |  |  |
|  |  | TLR-4: LYS153:HZ2 - **VACCINE: HIS295:NE2** |  | 1.94303 |
|  |  | TLR-4: LYS153:HZ3 - **VACCINE: HIS409:NE2** |  | 1.80943 |
|  |  | TLR-4: TYR183: HH - **VACCINE:ASP248:O** |  | 1.94326 |
|  |  | TLR-4: GLN187:HE22 - **VACCINE: LYS241:O** |  | 2.07613 |
|  |  | TLR-4: ARG256:HH21- **VACCINE: GLU298:OE1** |  | 2.00284 |
|  |  | TLR-4: ARG256:HH21- **VACCINE: GLU298:OE2** |  | 1.84045 |
|  |  | TLR-4: LYS263:HZ1 - **VACCINE: GLY372:O** |  | 1.94012 |
|  |  | TLR-4: LYS263:HZ2 - **VACCINE: THR371:O** |  | 1.80408 |
|  |  | TLR-4: ARG288:HH21-**VACCINE: GLN301:OE1** |  | 1.8007 |
|  |  | TLR-4: ARG337:HH11 - **VACCINE: GLY370:O** |  | 1.77532 |
|  |  | TLR-4: ARG337:HH21 - **VACCINE: GLY370:O** |  | 2.24268 |
|  |  | TLR-4: LYS360:HZ1 - **VACCINE: ASP375:OD1** |  | 2.41063 |
|  |  | TLR-4: LYS360:HZ2 - **VACCINE: ASP375:OD1** |  | 2.25357 |
|  |  | TLR-4: LYS360:HZ3 - **VACCINE: ASP375:OD1** |  | 1.92077 |
|  |  | TLR-4: LYS360:HZ3 - **VACCINE: ASP375:OD2** |  | 1.56806 |
|  |  | TLR-4: ARG380:HH11 - **VACCINE: TYR390:O** |  | 2.36639 |
|  |  | TLR-4: ARG380:HH12- **VACCINE: GLU395:OE1** |  | 1.59803 |
|  |  | TLR-4: LYS503:HZ1 - **VACCINE: ASP387:OD1** |  | 1.92289 |
|  |  | TLR-4: LYS503:HZ2 - **VACCINE: ASP387:OD1** |  | 1.59856 |
|  |  | TLR-4: LYS503:HZ2 - **VACCINE: ASP387:OD2** |  | 1.65563 |
|  |  | **VACCINE: LYS241:NZ** - TLR-4: GLN187:OE1 |  | 2.72149 |
|  |  | **VACCINE: LYS241:NZ** - TLR-4: ASP215:OD1 |  | 2.70695 |
|  |  | **VACCINE: LYS241:NZ** - TLR-4: ASP215:OD2 |  | 2.46475 |
|  |  | **VACCINE: ARG249:NE** - TLR-4: TYR185: OH |  | 2.87716 |
|  |  | **VACCINE: TYR252: OH** - TLR-4: GLU134:OE1 |  | 2.98153 |
|  |  | **VACCINE: GLN301:NE2** - TLR-4: GLU286:OE1 |  | 2.70377 |
|  |  | **VACCINE: TYR357: OH** - TLR-4: ASP264:OD1 |  | 3.01996 |
|  |  | **VACCINE: ARG364:NH1** - TLR-4: GLU265:OE1 |  | 2.68316 |
|  |  | **VACCINE: ARG364:NH2** - TLR-4: ASP264:OD2 |  | 2.71769 |
|  |  | **VACCINE: ARG364:NH2** - TLR-4: GLU265:OE1 |  | 2.85396 |
|  |  | **VACCINE: GLY366: N** - TLR-4: ASP180:OD2 |  | 3.07565 |
|  |  | **VACCINE: LYS382:NZ** - TLR-4: ASN407:O |  | 2.52038 |
|  |  | **VACCINE: LYS382:NZ** - TLR-4: HIS429:O |  | 2.5914 |
|  |  | **VACCINE: LYS398:NZ** - TLR-4: ASP377:OD1 |  | 2.46729 |
|  |  | **VACCINE: LYS398:NZ** - TLR-4: ASP377:OD2 |  | 2.61967 |
|  |  | **VACCINE: LYS398:NZ** - TLR-4: SER379: OG |  | 2.53901 |
|  |  | **VACCINE: ARG405:NE** - TLR-4: ASP208:OD2 |  | 3.07787 |
|  |  | **VACCINE: ARG405:NE** - TLR-4: THR231:OG1 |  | 2.88831 |
|  |  | **VACCINE: ARG405:NH2** - TLR-4: ASP208:OD2 |  | 2.64117 |
|  |  | **VACCINE: TYR406: OH** - TLR-4: GLU229:OE2 |  | 3.08856 |
|  |  | **VACCINE: ASN455:ND2** - TLR-4: VAL31:O |  | 2.84803 |
|  |  | TLR-4: LYS153:HZ2 - **VACCINE: HIS295:NE2** |  | 1.94303 |
|  |  | TLR-4: LYS153:HZ3 - **VACCINE: HIS409:NE2** |  | 1.80943 |
|  |  | TLR-4: TYR183: HH - **VACCINE:ASP248:O** |  | 1.94326 |
|  |  | TLR-4: GLN187:HE22 - **VACCINE: LYS241:O** |  | 2.07613 |
|  |  | TLR-4: ARG256:HH21-**VACCINE: GLU298:OE1** |  | 2.00284 |
|  |  | TLR-4: ARG256:HH21-**VACCINE: GLU298:OE2** |  | 1.84045 |
|  |  | TLR-4: LYS263:HZ1 - **VACCINE: GLY372:O** |  | 1.94012 |
|  |  | TLR-4: LYS263:HZ2 - **VACCINE: THR371:O** |  | 1.80408 |
|  |  | TLR-4: ARG288:HH21- **VACCINE: GLN301:OE1** |  | 1.8007 |
|  |  | TLR-4: ARG337:HH11 - **VACCINE: GLY370:O** |  | 1.77532 |
|  |  | TLR-4: ARG337:HH21 - **VACCINE: GLY370:O** |  | 2.24268 |
|  |  | TLR-4: LYS360:HZ1 - **VACCINE: ASP375:OD1** |  | 2.41063 |
|  |  | TLR-4: LYS360:HZ2 - **VACCINE: ASP375:OD1** |  | 2.25357 |
|  |  | TLR-4: LYS360:HZ3 - **VACCINE: ASP375:OD1** |  | 1.92077 |
|  |  | TLR-4: LYS360:HZ3 - **VACCINE: ASP375:OD2** |  | 1.56806 |
|  |  | TLR-4: ARG380:HH11 - **VACCINE: TYR390:O** |  | 2.36639 |
|  |  | TLR-4 ARG380:HH12 - **VACCINE: GLU395:OE1** |  | 1.59803 |
|  |  | TLR-4: LYS503:HZ1 - **VACCINE: ASP387:OD1** |  | 1.92289 |
|  |  | TLR-4: LYS503:HZ2 - **VACCINE: ASP387:OD1** |  | 1.59856 |
|  |  | TLR-4: LYS503:HZ2 - **VACCINE: ASP387:OD2** |  | 1.65563 |
|  |  | **VACCINE: LYS241:NZ** - TLR-4: GLN187:OE1 |  | 2.72149 |
|  |  | **VACCINE: LYS241:NZ** - TLR-4: ASP215:OD1 |  | 2.70695 |
|  |  | **VACCINE: LYS241:NZ** - TLR-4: ASP215:OD2 |  | 2.46475 |
|  |  | **VACCINE: ARG249:NE** - TLR-4: TYR185: OH |  | 2.87716 |
|  |  | **VACCINE: TYR252: OH** - TLR-4: GLU134:OE1 |  | 2.98153 |
|  |  | **VACCINE: THR286:OG1** - TLR-4: ASP83:OD2 |  | 2.39154 |
|  |  | **VACCINE: GLN301:NE2** - TLR-4: GLU286:OE1 |  | 2.70377 |
|  |  | **VACCINE: TYR357: OH** - TLR-4: ASP264:OD1 |  | 3.01996 |
|  |  | **VACCINE: ARG364:NH1** - TLR-4: GLU265:OE1 |  | 2.68316 |
|  |  | **VACCINE: ARG364:NH2** - TLR-4: ASP264:OD2 |  | 2.71769 |
|  |  | **VACCINE: ARG364:NH2** - TLR-4: GLU265:OE1 |  | 2.85396 |
|  |  | **VACCINE: GLY366: N** - TLR-4: ASP180:OD2 |  | 3.07565 |
|  |  | **VACCINE: LYS382:NZ** - TLR-4: ASN407:O |  | 2.52038 |
|  |  | **VACCINE: LYS382:NZ** - TLR-4: HIS429:O |  | 2.5914 |
|  |  | **VACCINE: LYS398:NZ** - TLR-4: ASP377:OD1 |  | 2.46729 |
|  |  | **VACCINE: LYS398:NZ** - TLR-4: ASP377:OD2 |  | 2.61967 |
|  |  | **VACCINE: LYS398:NZ** - TLR-4: SER379: OG |  | 2.53901 |
|  |  | **VACCINE: ARG405:NE** - TLR-4: ASP208:OD2 |  | 3.07787 |
|  |  | **VACCINE: ARG405:NE** - TLR-4: THR231:OG1 |  | 2.88831 |
|  |  | **VACCINE: ARG405:NH2** - TLR-4: ASP208:OD2 |  | 2.64117 |
|  |  | **VACCINE: TYR406: OH** - TLR-4: GLU229:OE2 |  | 3.08856 |
|  |  | **VACCINE: ASN455:ND2** - TLR-4: VAL31:O |  | 2.84803 |
|  |  | TLR09:TRP47: HE1 - **Vaccine: ASP11:OD1** |  | 1.99832 |
|  |  | TLR09:ARG74: HH11 - **Vaccine: ASP23:OD1** |  | 1.83771 |
|  |  | TLR09:ARG74: HH21 - **Vaccine: ASP23:OD1** |  | 2.09777 |
|  |  | TLR09:ARG74: HH21 - **Vaccine: ASP23:OD2** |  | 2.05669 |
|  |  | TLR09:LYS181: HZ1 - **Vaccine: GLU8:OE1** |  | 1.7386 |
| MNEV | **TLR-9** | TLR09:LYS181: HZ2 - **Vaccine: GLU8:OE1** |  | 2.42305 |
|  |  | TLR09:LYS181: HZ2 - **Vaccine: GLU8:OE2** |  | 1.74107 |
|  |  | TLR09:LYS348: HZ1 - **Vaccine: SER86:O** |  | 2.16474 |
|  |  | TLR09:ARG482: HH11 - **Vaccine: ASP72:OD1** |  | 2.14165 |
|  |  | TLR09:ARG482: HH21 - **Vaccine: ASP72:OD1** |  | **1.8735** |
|  |  | TLR09:ARG482: HH21 - **Vaccine: ASP72:OD2** |  | 1.8362 |
|  |  | TLR09:HIS735: HD1 - **Vaccine: GLU115:OE1** |  | 1.98664 |
|  |  | TLR09:SER760: HG - **Vaccine: GLU115:OE2** |  | 1.99153 |
|  |  | TLR09:ARG800: HH11 - **Vaccine: GLU29:OE1** |  | 1.76825 |
|  |  | TLR09:ARG800: HH21 - **Vaccine: GLU29:OE1** |  | 2.43292 |
|  |  | TLR09:ARG800: HH21 - **Vaccine: GLU29:OE2** |  | 1.89206 |
|  |  | **Vaccine: MET1:H** - TLR09:PRO262:O |  | 2.29128 |
|  |  | **Vaccine: LYS73:HZ1** - TLR09:SER423: OG |  | 1.70022 |
|  |  | **Vaccine: LYS73:HZ2** - TLR09:GLN399: OE1 |  | 1.69435 |
|  |  | **Vaccine: LYS73:HZ3** - TLR09:ASP421: OD1 |  | 2.08904 |
|  |  | **Vaccine: LYS73:HZ3** - TLR09:ASP421: OD2 |  | 1.82547 |
|  |  | **Vaccine: LYS79:HZ3** - TLR09:ASP424: OD1 |  | 1.80256 |
|  |  | **Vaccine: ARG82:HH21** - TLR09:ASP259:O |  | 1.77958 |
|  |  | **Vaccine: ARG82:HH22** - TLR09:TYR345: OH |  | 1.66225 |
|  |  | TLR3:ARG31: HE - **Vaccine: ILE445:O** |  | 1.92293 |
|  |  | TLR3:ARG31: HH11 - **Vaccine: TYR293: OH** |  | 2.18218 |
|  |  | TLR3:ARG31: HH21 - **Vaccine: TYR293: OH** |  | 1.77134 |
|  |  | TLR3:ARG31: HH22 - **Vaccine: ILE445:O** |  | 1.7999 |
|  |  | TLR3:TYR32: HH - **Vaccine: ASP289:OD2** |  | 2.14168 |
| MNEV | **TLR3** | TLR3:GLN278: HE21 - **Vaccine: ASP267:OD2** |  | 2.12652 |
|  |  | TLR3:GLN278: HE22 - **Vaccine: CYS264:O** |  | 1.92778 |
|  |  | TLR3:TYR302: HH - **Vaccine: THR265:OG1** |  | 1.8778 |
|  |  | TLR3:THR411: HG1 - **Vaccine: GLN426:OE1** |  | 1.85788 |
|  |  | TLR3:LYS416: HZ1 - **Vaccine: HIS467:NE2** |  | 1.81092 |
|  |  | TLR3:ARG434: HH11 - **Vaccine: SER344:O** |  | 1.90431 |
|  |  | TLR3:ARG484: HH11 - **Vaccine: VAL336:O** |  | 1.84353 |
|  |  | TLR3:ARG484: HH21 - **Vaccine: ARG337:O** |  | 1.73288 |
|  |  | TLR3:ARG484: HH22 - **Vaccine: ARG340:O** |  | 1.80265 |
|  |  | TLR3:ARG489: HH12 - **Vaccine: HIS472:NE2** |  | 2.28453 |
|  |  | TLR3:ARG613: HH11 - **Vaccine: GLU395:OE1** |  | 2.0522 |
|  |  | TLR3:ARG613: HH11 - **Vaccine: GLU395:OE2** |  | 2.32248 |
|  |  | TLR3:ARG613: HH21 - **Vaccine: GLU395:OE2** |  | 1.81961 |
|  |  | TLR3:SER614: HG - **Vaccine: ASP387:OD2** |  | 1.94886 |
|  |  | **Vaccine: LYS269:HZ2** - TLR3:TYR326: OH |  | 1.97913 |
|  |  | **Vaccine: LYS269:HZ3** - TLR3:TYR326: OH |  | 2.29645 |
|  |  | **Vaccine: LYS269:HZ3** - TLR3:TYR359: OH |  | 1.71838 |
|  |  | **Vaccine: LYS296:HZ2** - TLR3:TYR32: OH |  | 1.66288 |
|  |  | **Vaccine: ARG334:HH12** - TLR3:ASP437: OD1 |  | 1.79657 |
|  |  | **Vaccine: ARG334:HH22** - TLR3:ASP437: OD2 |  | 1.82492 |
|  |  | **Vaccine: ARG337:HH11** - TLR3:GLU460: OE2 |  | 1.78315 |
|  |  | **Vaccine: ARG337:HH12** - TLR3:ASP437: OD2 |  | 1.91642 |
|  |  | **Vaccine: ARG337:HH22** - TLR3:ASP437: OD1 |  | 1.97754 |
|  |  | **Vaccine: ARG337:HH22** - TLR3:ASP437: OD2 |  | 1.98642 |
|  |  | **Vaccine: ARG340:HH12** - TLR3:GLU533: OE1 |  | 1.95119 |
|  |  | **Vaccine: ARG340:HH12** - TLR3:GLU533: OE2 |  | 1.92055 |
|  |  | **Vaccine: ARG340:HH22** - TLR3:GLU533: OE1 |  | 1.87025 |
|  |  | **Vaccine: ARG340:HH22** - TLR3:GLU533: OE2 |  | 2.21064 |
|  |  | **Vaccine: ARG385:HH11** - TLR3:ASP536: OD1 |  | 2.20122 |
|  |  | **Vaccine: ARG385:HH11** - TLR3:ASP536: OD2 |  | 1.97728 |
|  |  | **Vaccine: ARG385:HH12** - TLR3:ASP512: OD2 |  | 2.02167 |
|  |  | **Vaccine: ARG385:HH22** - TLR3:ASP512: OD2 |  | 1.78468 |
|  |  | **Vaccine: ARG449:HH11** - TLR3:ASN57: OD1 |  | 1.80853 |
|  |  | **Vaccine: ARG449:HH12** - TLR3:ASP36: OD1 |  | 1.93472 |
|  |  | **Vaccine: ARG449:HH12** - TLR3:ASP36: OD2 |  | 2.09027 |
|  |  | **Vaccine: ARG449:HH22** - TLR3:ASP36: OD2 1.76074 | | |
